# Review on fractional vortex beam

**DOI:** 10.1515/nanoph-2021-0616

**Published:** 2021-12-19

**Authors:** Hao Zhang, Jun Zeng, Xingyuan Lu, Zhuoyi Wang, Chengliang Zhao, Yangjian Cai

**Affiliations:** School of Physical Science and Technology, Soochow University, Suzhou 215006, China; School of Physics and Electronics, Shandong Provincial Engineering and Technical Center of Light Manipulations & Shandong Provincial Key Laboratory of Optics and Photonic Devices, Shandong Normal University, Jinan 250014, China

**Keywords:** fractional vortex beam, orbital angular momentum, phase step, singular optics, structured light, topological charge jump

## Abstract

As an indispensable complement to an integer vortex beam, the fractional vortex beam has unique physical properties such as radially notched intensity distribution, complex phase structure consisting of alternating charge vortex chains, and more sophisticated orbital angular momentum modulation dimension. In recent years, we have noticed that the fractional vortex beam was widely used for complex micro-particle manipulation in optical tweezers, improving communication capacity, controllable edge enhancement of image and quantum entanglement. Moreover, this has stimulated extensive research interest, including the deep digging of the phenomenon and physics based on different advanced beam sources and has led to a new research boom in micro/nano-optical devices. Here, we review the recent advances leading to theoretical models, propagation, generation, measurement, and applications of fractional vortex beams and consider the possible directions and challenges in the future.

## 1 Introduction

Vortex beam specifically refers to a type of beam carrying helical phase [1], [2], [3] that is formed by the spiral rotation of the wave front along the direction of the optical axis, and can be described quantitatively by the phase factor exp(i*lθ*), where *l* and *θ* represent the topological charge (TC) and azimuth angle, respectively. The manipulation of the vortex phase in the optical field has led to the emergence of a new subject, namely, singularity optics [1, 4, 5]. In 1992, Allen et al. pointed out that a vortex beam carries an orbital angular momentum (OAM) of *lħ* per photon (*ħ* is the reduced Planck constant) and revealed a new connection between macroscopic optics and quantum effects [6]. Obviously, the OAM is the eigenvalue and is robust [7] that determines the OAM value carried by each photon. Compared with the traditional plane wave and spherical wave, vortex beams have distinct features of helical phase front and doughnut intensity structures owing to the center phase singularity [8]. The unique physical properties of vortex beams facilitate a variety of applications such as optical communication [9], [10], [11], particle manipulation [12], [13], [14], [15], optical imaging [16], [17], [18], [19], [20], quantum information [21], [22], [23], [24], astronomy [25], [26], [27], optical detection [28], [29], [30], medical diagnosis [31], and many other applications in different fields [3]. To improve the practical application of the vortex beam, an increasing number of new methods for generating the vortex beam and detecting TC have been proposed in the past five years [32], [33], [34], [35], [36], [37], [38], [39], [40] that shows that vortex beams have stimulated innovation in various fields. Moreover, further research on vortex beam regulation is expected to promote the birth of new physical phenomena and scientific applications, and has important scientific significance.

In most studies related to vortices, the value of TC is merely restricted as an integer where the helical phase has a 2*l*π step. In fact, the value of TC can also be a noninteger (the phase step is not an integer multiple of 2π), and a vortex beam with a noninteger TC is termed as a fractional vortex beam [41], [42], [43]. In contrast to the integer-order vortex beam, the phase appears as a discontinuity along the phase step, and the annular intensity ring is broken as a radial dark opening (or low intensity gap). Note that the radial dark opening and the phase circulations of the open vortex beam are completely different from those of fractional vortex beam, although the open vortex beam and the fractional vortex beam both have the radial dark opening structure [44]. In 2004, Berry theoretically studied the vortex structure of a beam with a fractional phase step in detail, and mentioned that the fractional vortex beam can be expressed as a superposition of a series of integer vortices [45]. In other words, a vortex beam with fractional TC could be decomposed into a Fourier series of integer vortex beams with different intensity weights. When TC *l* is a half integer, an infinite chain of alternating-strength vortices appears at the position of the phase step discontinuity, and vanish when TC *l* is larger or smaller than a half integer. This property of the fractional vortex was further elucidated in Hilbert’s Hotel phenomenon [46]. It is worth noting that the OAM of each photon can carry integer or noninteger values in units of *ħ* [47, 48]. The significant characteristic of the fractional vortex beam is that it cannot propagate stably in free space. However, in other words, it exhibits a rich evolutionary process that induces complex amplitude and phase structures and increases the regulatory degree of freedom [45, 46, 49], [50], [51], [52]. More specifically, based on its intrinsic characteristics, the fractional vortex beam can be classified as a fractional Gaussian vortex beam [41, 53], fractional Bessel–Gaussian (BG) beams [51, 54], fractional Laguerre–Gaussian (LG) beams [55, 56], perfect fractional vortex beams [57, 58], fractional elliptic vortex beams [59, 60], and partially coherent fractional vortex beams [61, 62].

In recent years, fractional vortex beams have attracted enormous attention in the field of light manipulation owing to their unusual properties. First, the interaction between light and matter is the most intuitive mechanism to demonstrate the potential applications of fractional vortex beams. Compared with the integer vortex beam that only realizes a rotation on the light ring, a fractional vortex beam possesses a unique intensity distribution that can realize cell sorting [63] or precise control of the cell orientation [64]. It is always an enormous challenge to significantly increase the information capacity of optical communication systems, where the OAM modes are mutually orthogonal and can be regarded as a new degree of freedom to address this problem [65]. Thus, in optical communication systems, the fractional vortex beam with continuous integer and noninteger OAM states [47, 48] can overcome the limitation of aperture size and expand the communication capacity [66, 67]. Another practical application of fractional vortex beam is optical imaging. It has been verified that a beam carrying an OAM can realize image edge enhancement [68, 69]. Compared with a regular vortex beam that only achieves an isotropic edge enhancement, the fractional vortex beam can realize anisotropic edge enhancement [70, 71]. Furthermore, the fractional vortex beam can effectively resist noise influence and realize high-resolution imaging in radar imaging systems [72]. In quantum optics, the fractional vortex beam can facilitate the realization of spatial entanglement in an infinite-dimensional subspace [73] due to the fact that fractional-OAM states are coherent superpositions of an infinite number of LG states with integer OAM. Moreover, the fractional vortex beam can also be regarded as a phase object in quantum digital spiral imaging to demonstrate the high-dimensional nature of the associated quantum OAM channels [74].

Owing to the extensive research interests and potential applications of fractional vortex beams, researchers need a review paper urgently to give a comprehensive and thorough introduction of fractional vortex beams. Hence, we focused on some of the landmark advances of fractional vortex beams in this review: the basic theory, propagation properties, experimental generation, measurement, and applications. For more information on the introduction, generation, properties, and applications of vortex beams with integer TC, the readers can refer to the previous classical review papers [3, 8, 12, 75], [76], [77], [78]. This review includes a general introduction (Section 1), the theoretical models of six categories of fractional vortex beams ranging from fully coherent to partially coherent (Section 2), propagation properties (Section 3), classical experimental generation methods of different categories of fractional vortex beams (Section 4), measurement of the TC and OAM (Section 5), and applications in optical manipulation, optical communication, optical imaging, and quantum entanglement (Section 6). Finally, we summarize the research on fractional vortex beam and its future development.

## 2 Theoretical models of fractional vortex beams

From the perspective of coherence, we categorize fractional vortex beams into fully coherent fractional vortex beams and partially coherent fractional vortex beams that are modeled based on specific electric field distributions and statistical properties [e.g., cross spectral density (CSD) function], respectively.

### 2.1 Theoretical models of fully coherent fractional vortex beams

A fully coherent beam is typically characterized by its complex amplitude. Without considering the initial phase, the electric field distribution of a fully coherent vortex beam at the source plane (*z* = 0) can be expressed as [78]

(1) E(r,θ)=A(r)exp(ilθ),

where ***r*** and *θ* = arctan(*y*/*x*) denote the position vector and azimuthal coordinates in the source plane, respectively. *A*(***r***) is the amplitude, and *l* denotes the TC that can be an arbitrary value, both integral and fractional. By choosing a fractional value of *l*, the beam source, whose electric field is given by Eq. (1), is termed a fractional vortex beam. Various fractional vortex beams have been proposed by varying the amplitude and phase term, including fractional Gaussian vortex beams, fractional BG beams, fractional LG beams, perfect fractional vortex beams, and fractional elliptic vortex beams.

#### 2.1.1 Fractional Gaussian vortex beam

**Figure 1:**
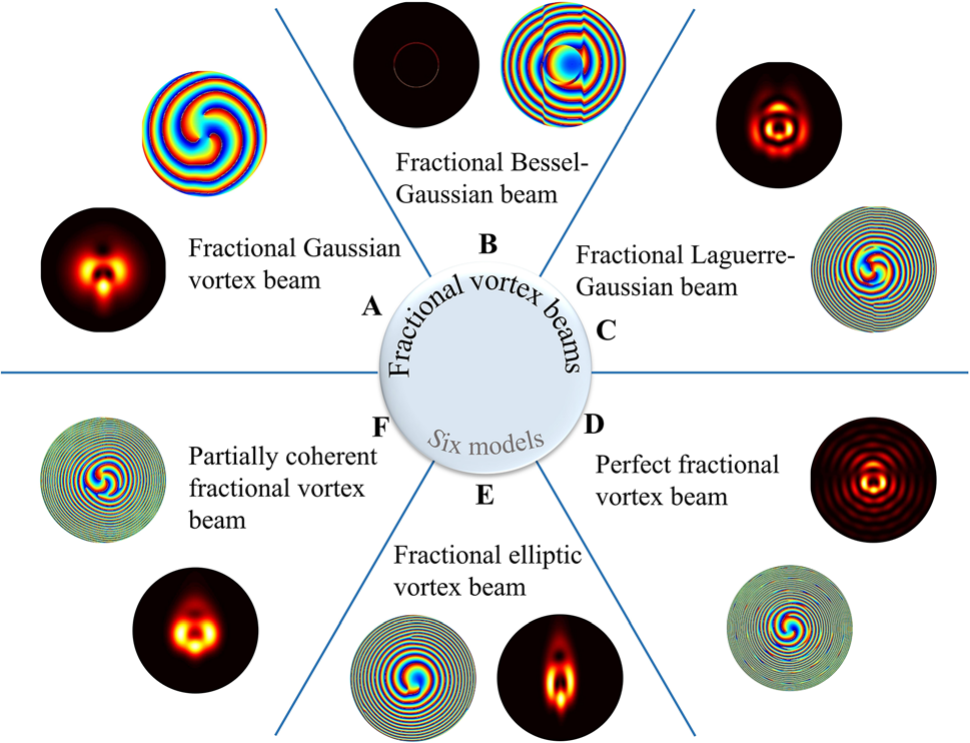
Intensity and phase patterns of the theoretical models of six categories of fractional vortex beams (TC *l* = 2.5) at the focal plane. (A) Fractional Gaussian vortex beam. (B) Fractional BG beam. (C) Fractional LG beam with *p* = 1. (D) Perfect fractional vortex beam. (E) Fractional elliptic vortex beam. (F) Partially coherent fractional vortex beam with spatial coherence width *σ*_g_ = 2*w*_0_.

A Gaussian beam with a finite beam width is usually preferred in practice over a plane wave with infinite energy. The fractional phase azimuthal variation hosted within a Gaussian beam can be expressed as [79, 80]

(2) $$E_{\text{GFV}}\left(r,\theta \right)=\exp\left(-\frac{r^{2}}{w_{0}^{2}}\right)\exp\left(il\theta \right)\text{,}$$

where *w*_0_ is the waist radius of the Gaussian beam, and *l* is a real fractional value. Figure 1A illustrates the intensity and phase patterns of the fractional Gaussian vortex beam at the focal plane. It should be noted that for the fractional Gaussian vortex beam represented by Eq. (2), we usually decompose a fractional vortex phase term into the bases of the integer vortex phase term [45, 46]:

(3) $$\exp\left(il\theta \right)=\frac{\exp\left(i\pi l\right)\sin\left(\pi l\right)}{\pi }\sum_{n=-\infty }^{\infty }\frac{\exp\left(in\theta \right)}{l-n}\text{,}$$

where *n* is an integer. In particular, by modifying the transmission function used to generate the vortex phase term, we can generate multi-ramp fractional Gaussian vortex beams with rich and varied TC jump characteristics [46, 81], [82], [83].

#### 2.1.2 Fractional Bessel–Gaussian beam

Similar to the definition of the fractional Gaussian vortex beam, the expression of the electric field of the fractional BG beam can be obtained by simply adjusting the TC of the higher-order BG beam to a fractional value [49, 84]:

(4) $$E_{\text{FB}}\left(r,\theta \right)=\exp\left(-\frac{r^{2}}{w_{0}^{2}}\right)J_{l}\left(k_{r}r\right)\exp\left(il\theta \right)\text{,}$$

where *J*_*l*_ is the *l* th-order Bessel function of the first type, and *k*_*r*_ is the radial wave number. The intensity and phase patterns of the fractional BG beam at the focal plane are shows in Figure 1B. Although this definition is widely used, considering that Eq. (4) with fractional *l* is not an appropriate solution to the Helmholtz equation [85], a new model of the fractional BG beam based on the inverse Fourier transform of the angular spectrum is proposed that can be expressed in terms of the *n*th-order Bessel beams with the same transverse frequency [86, 87]:

(5) $$E_{\text{NFB}}\left(r,\theta \right)=\sum_{n=-\infty }^{\infty }\frac{i^{l-n}\;\sin\left[\pi \left(n-l\right)\right]}{\pi \left(n-l\right)}J_{n}\left(k_{0}r\right)\exp\left(in\theta \right)\text{,}$$

where *k*_0_ is the transverse wave number of the beam. When *l* tends to integer *n*, the term sin[π(*n* − *l*)]/π(*n* − *l*) tends to unity, and Eq. (5) reduces simply to the *n*th-order Bessel beams *J*_*n*_(*k*_0_ *r*)exp(i*nθ*). To distinguish between these two models, we call the first one the conventional fractional BG model.

#### 2.1.3 Fractional Laguerre–Gaussian beam

LG beams are the earliest reported vortex beams carrying OAM. Therefore, by choosing a fractional TC, the corresponding fractional LG beam with a zero radial index has also been researched [88].

(6) $$E_{\text{FLG}}\left(r,\theta \right)=A_{1}{\left(\frac{r}{w_{0}}\right)}^{\left|l\right|}L_{p}^{\left|l\right|}\left(\frac{2r^{2}}{w_{0}^{2}}\right)\exp\left(-\frac{r^{2}}{w_{0}^{2}}\right)\exp\left(il\theta \right)\text{,}$$

where *A*_1_ is a normalization constant, $L_{p}^{\left|l\right|}$ is the Laguerre polynomial. *l* and *p* are the azimuthal (TC) and radial indices, respectively. Figure 1C shows the intensity and phase patterns of the fractional LG beam at the focal plane. When *p* tends to zero, Eq. (6) is reduced to the simplest fractional LG beam described in ref. [89]. In contrast to the definition above, some researchers determined the field superposition resulting from a nonlinear process in terms of LG beams with both arbitrary integer radial and angular indices that are orthonormal. The resulting fractional LG field can be obtained as [55, 90], [91], [92]

(7) $$E_{\text{FLG}}\left(r,\theta \right)=\sum_{n=-\infty }^{\infty }C_{n}u_{p}^{n}\text{,}$$

(8) $$C_{n}=\exp\left(-i\mu \theta \right)\frac{i\;\exp\left[i\left(l-n\right)\theta_{0}\right]}{2\pi \left(l-n\right)}\exp\left[i\left(l-n\right)\theta \right]\left[1-\exp\left(i2\pi \mu \right)\right]\text{,}$$

(9) $$u_{p}^{n}=\frac{C_{np}}{\sqrt{w_{0}}}{\left(\frac{\sqrt{2}r}{w_{0}}\right)}^{\left|n\right|}\;\exp\left(-\frac{r^{2}}{w_{0}^{2}}\right)L_{p}^{\left|n\right|}\left(\frac{2r^{2}}{w_{0}^{2}}\right)\exp\left(in\theta \right)\text{,}$$

where *l* and *μ* are the fractional TC of the beam and the fractional part of *l*, respectively. The normalization constants of the integer LG beams are expressed as $C_{np}=\sqrt{2p!/\left[\pi \left(\left|n\right|+p\right)!\right]}$ .

In addition, with the assistance of the fractional application of the rising and lowering operators, a new fractional-order solution of the paraxial wave equation, termed fractional elegant LG beam, is proposed as a superposition of beams with integer angular indices [56, 93]:

(10) $$E_{\text{FELG}}\left(r,\theta \right)={\left(-i\right)}^{l}\sum_{n=-\infty }^{\infty }{\left(-1\right)}^{n}\frac{\sin\left[\pi \left(n-l\right)\right]}{\pi \left(n-l\right)}E_{p,n}\left(r,\theta \right)\text{,}$$

with

(11) Ep,n(r,θ)=i2p+lΓ(η+|n|+1)Γ(|n|+1)(2w0)2η+|n|(rw0)|n|2π1w0exp(−r2w02)×F11(−η,|n|+1,rw0)exp(inθ),

where *η* = *p* + (*l* − *n*)/2, Γ(·), and _1_ *F*_1_(·) are the gamma function and confluent hypergeometric function, respectively.

#### 2.1.4 Perfect fractional vortex beam

We recall the perfect vortex beam with an integer TC generation process that can be easily obtained through the Fourier transformation of a high-order BG beam [94]. Using a convex lens with a focal length *f*, to conduct the Fourier transformation of Eq. (4), the perfect fractional vortex beam can be produced at the recording plane that can be expressed as [57, 59].

(12) $$E_{\text{FPV}}\left(\rho ,\varphi \right)=\frac{w_{0}i^{l-1}}{w_{1}}\exp\left(il\varphi \right)\exp\left[-\frac{{\left(\rho -R\right)}^{2}}{w_{1}^{2}}\right]\text{,}$$

where *w*_1_ = 2*f*/*kw*_0_ is the beam waist at the focus that should be a small value here to achieve a perfect vortex beam approximation, and *R* is a constant that determines the radius of the perfect fractional vortex beam. (*ρ*, *φ*) denotes the polar coordinates at the recording plane. The intensity and phase patterns of the perfect fractional vortex beam at the focal plane are depicted in Figure 1D. Sometimes, for simplicity, Eq. (12) can be written in the form of a constant amplitude [94].

#### 2.1.5 Fractional elliptic vortex beam

Compared with vortex beams with circular symmetry, elliptical vortex beams with asymmetric distributions have unique advantages in particle trapping. With the concept of coordinate transformation, the field of the fractional elliptic vortex beam at the aperture plane is expressed as [60]

(13) $$E_{\text{FEV}}\left(x,y\right)=\exp\left[-\frac{x^{2}+{\left(\epsilon y\right)}^{2}}{w_{0}^{2}}\right]{\left[\frac{\sqrt{x^{2}+{\left(\epsilon y\right)}^{2}}}{w_{0}}\right]}^{\left|l\right|}\;\exp\left[il\cdot \text{arctan}\left(\frac{\epsilon y}{x}\right)\right]\text{,}$$

where *ε* and arctan(*ε*·*y*/*x*) denote the elliptic parameter and the azimuth angle of the fractional elliptic vortex beam, respectively, and *x* and *y* are the Cartesian coordinates. Figure 1E demonstrates the intensity and phase patterns of the fractional elliptic vortex beam at the focal plane. When *ε* is equal to 1, the beam source represented by Eq. (13) reduces to a fractional LG beam, as shown in [55, 89]. Based on the concept of elliptical vortex beam generation, we can obtain a perfect fractional elliptic vortex beam by stretching the fractional BG beam to an elliptic BG beam and subsequently applying a Fourier transform operation [59, 95]:

(14) $$E_{\text{FEPV}}\left(\rho ,\varphi \right)=\frac{w_{0}i^{l-1}}{w_{2}}\exp\left[il\cdot \arctan\left(\frac{\epsilon y}{x}\right)\right]\exp\left[-\frac{{\left(\rho -\sqrt{x^{2}+{\left(\epsilon y\right)}^{2}}\right)}^{2}}{w_{2}^{2}}\right]\text{,}$$

where *w*_2_ = *εw*_1_, and *ε* is a positive scaling factor. When *ε* is equal to 1, the beam source represented by Eq. (14) is reduced to the model described by Eq. (12). In addition, fractional vortex phases are also introduced into Airy beams and vector light fields [95], [96], [97], [98], [99], [100], [101] that are not detailed here.

### 2.2 Theoretical models of partially coherent fractional vortex beams

Unlike fully coherent optical vortex beams, it is generally accepted that a partially coherent beam can be characterized by its statistical properties. The CSD function of a partially coherent vortex beam at the source plane is defined as a two-point correlation function [102]:

(15) $$W\left(r_{1},r_{2}\right)=〈E\left(r_{1}\right)E^{*}\left(r_{2}\right)〉\text{,}$$

where ***r***_1_ and ***r***_2_ are the position vectors at the source plane, the angular brackets denote an ensemble average, and the asterisk denotes the complex conjugate. By substituting the electric field distribution of fully coherent fractional vortex beams into Eq. (15), we can obtain the theoretical model of partially coherent fractional vortex beams:

(16) $$W\left(r_{1},r_{2}\right)=A\left(r_{1}\right)A\left(r_{2}\right)g\left(r_{1}-r_{2}\right)\exp\left[il\left(\theta_{1}-\theta_{2}\right)\right]\text{,}$$

where *g*(***r***_1_ − ***r***_2_) denotes the correlation function between two points ***r***_1_ and ***r***_2_. In 2018, our group introduced fractional TC into a partially coherent light field and generated a new type of partially coherent vortex beam with fractional TC named partially coherent fractional vortex beam, whose CSD function is expressed as [61]

(17) $$W_{\text{FV}}\left(r_{1},r_{2}\right)={\left(\frac{2r_{1}r_{2}}{w_{0}^{2}}\right)}^{l}\;\exp\left[-\frac{r_{1}^{2}+r_{2}^{2}}{w_{0}^{2}}\right]\exp\left[-il\left(\theta_{1}-\theta_{2}\right)\right]\left[-\frac{{\left(r_{1}-r_{2}\right)}^{2}}{2\sigma_{\text{g}}^{2}}\right]\text{,}$$

where *σ*_g_ is the spatial coherence width and the intensity and phase patterns of the partially coherent fractional vortex beam at the focal plane are shown in Figure 1F. If *σ*_g_ tends to infinity, Eq. (17) is reduced to the fractional LG beam with *p* = 0 [88]. In particular, as a natural extension of the scalar partially coherent fractional vortex beams, we have proposed a partially coherent radially polarized fractional vortex beam by considering the polarization characteristics [62].

## 3 Propagation of fractional vortex beams

**Figure 2:**
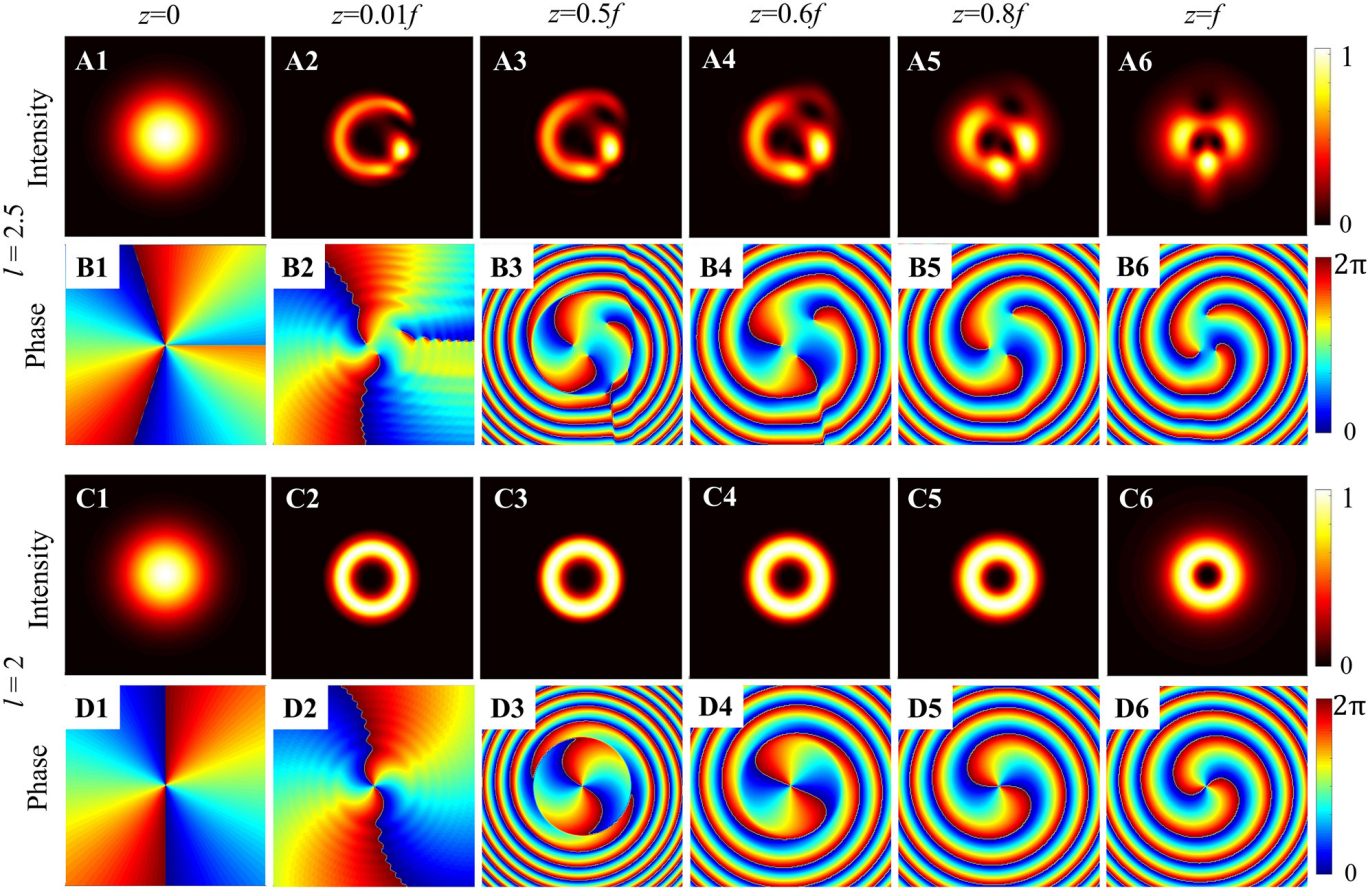
Intensity and phase evolutions of fractional and integer Gaussian vortex beams with different *l* focused by a thin lens at several propagation distances. (A1–A6) Intensity patterns and (B1–B6) phase patterns for fractional Gaussian vortex beams with TC *l* = 2.5. (C1–C6) Intensity patterns and (D1–D6) phase patterns for integer Gaussian vortex beams with TC *l* = 2.

To explain the propagation characteristics of the fractional vortex beam, we first take the Gaussian vortex beam model as an example and present the comparison diagram of propagation evolution between integer and fractional vortex beams, as shown in Figure 2. It is interesting to determine whether it is a fractional Gaussian vortex beam or an integer Gaussian vortex beam; the intensity at the source plane (*z* = 0) exhibits a Gaussian profile and the vortex characteristics are not visible, but once propagated, the vortex properties are reflected in the intensity pattern. In contrast to the integer vortex beam (see Figure 1C1–C6 and D1–D6) that has a circular symmetric structure, the fractional vortex beam has an opening gap in the intensity ring encompassing the dark core, and the radial opening can rotate with propagation, as demonstrated in Figure 2A2–A6. In fact, the closer the TC is to the half-integral value, the larger the gap, which is not shown here [63]. By comparing the phase evolution of the fractional vortex beam and integer vortex beam, it is observed that the phase singularities of the former are separated, as shown in Figure 2B1–B2, during the transmission process, and new phase singularities are generated and annihilated along with them, as illustrated in Figure 2B2–B4 that is also the fundamental cause of TC jump and OAM oscillation [45], [46], [47].

**Figure 3:**
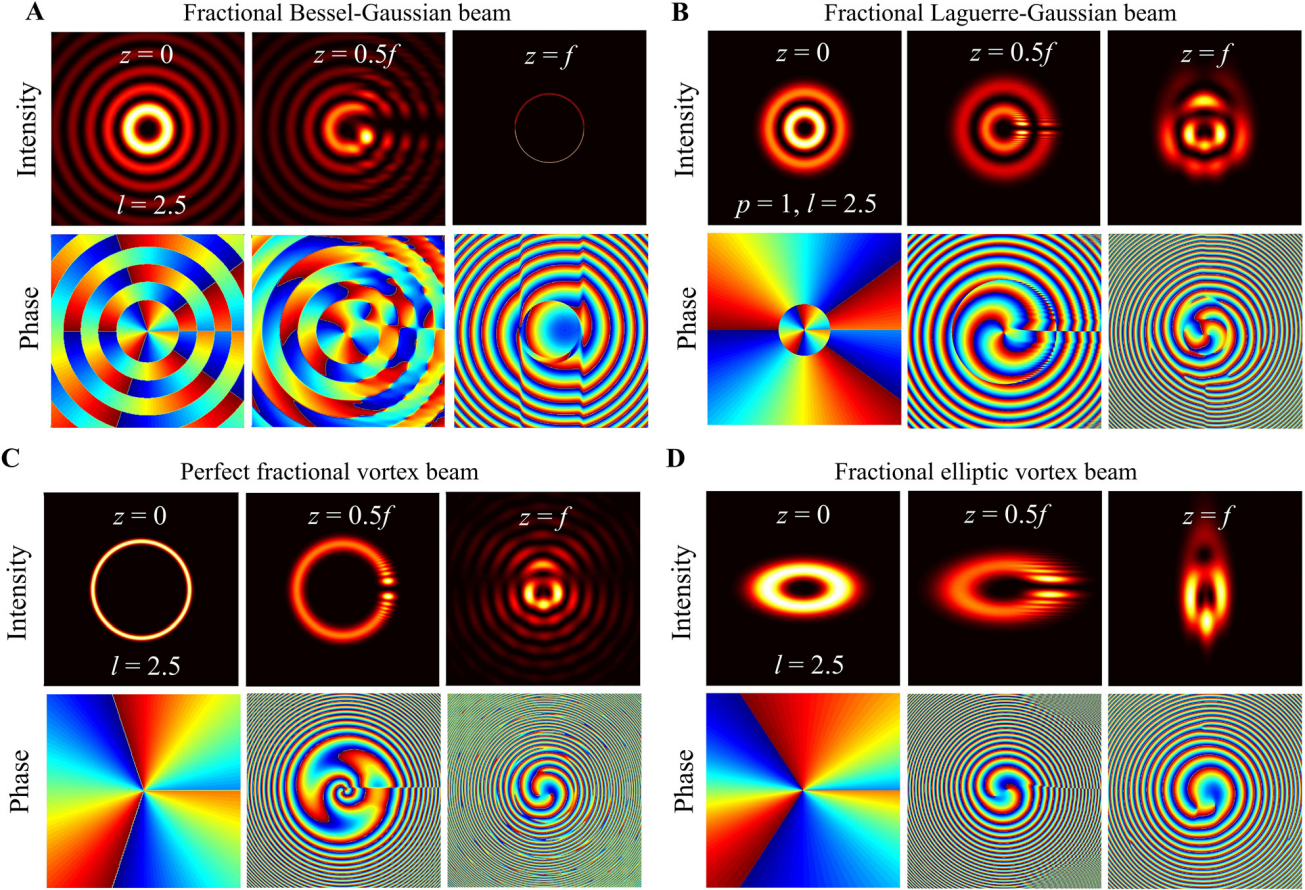
Intensity and phase evolutions of different types of fractional vortex beam for *l* = 2.5 during propagation. (A) Fractional BG beam, (B) fractional LG beams with different *p* = 1, (C) perfect fractional vortex beam, and (D) fractional elliptic vortex beam.

The intensity and phase evolution of other fully coherent fractional vortex beam models are shown in Figure 3. They also maintain radial gap characteristics and complex phase evolution characteristics in the transmission process, particularly exhibiting vortex characteristics (i.e., dark core) at the source plane, excluding radial opening. Moreover, partially coherent fractional vortex beams were proposed, and their unique propagation evolution was studied based on coherent modulation. This shows that the opening gap of the intensity pattern and the rotation of the beam spot disappear gradually, and the CSD distribution becomes more symmetric and more recognizable with a decrease in the spatial coherence width [61, 62]. In conclusion, fractional vortex beams exhibit unique optical properties during propagation, such as splitting, generation and annihilation of phase singularities, beam shaping and rotation, TC jump, and OAM oscillations that are considerably different from integer vortex beams, and we will focus on these in the following part of this paper.

### 3.1 Stability of propagation

**Figure 4:**
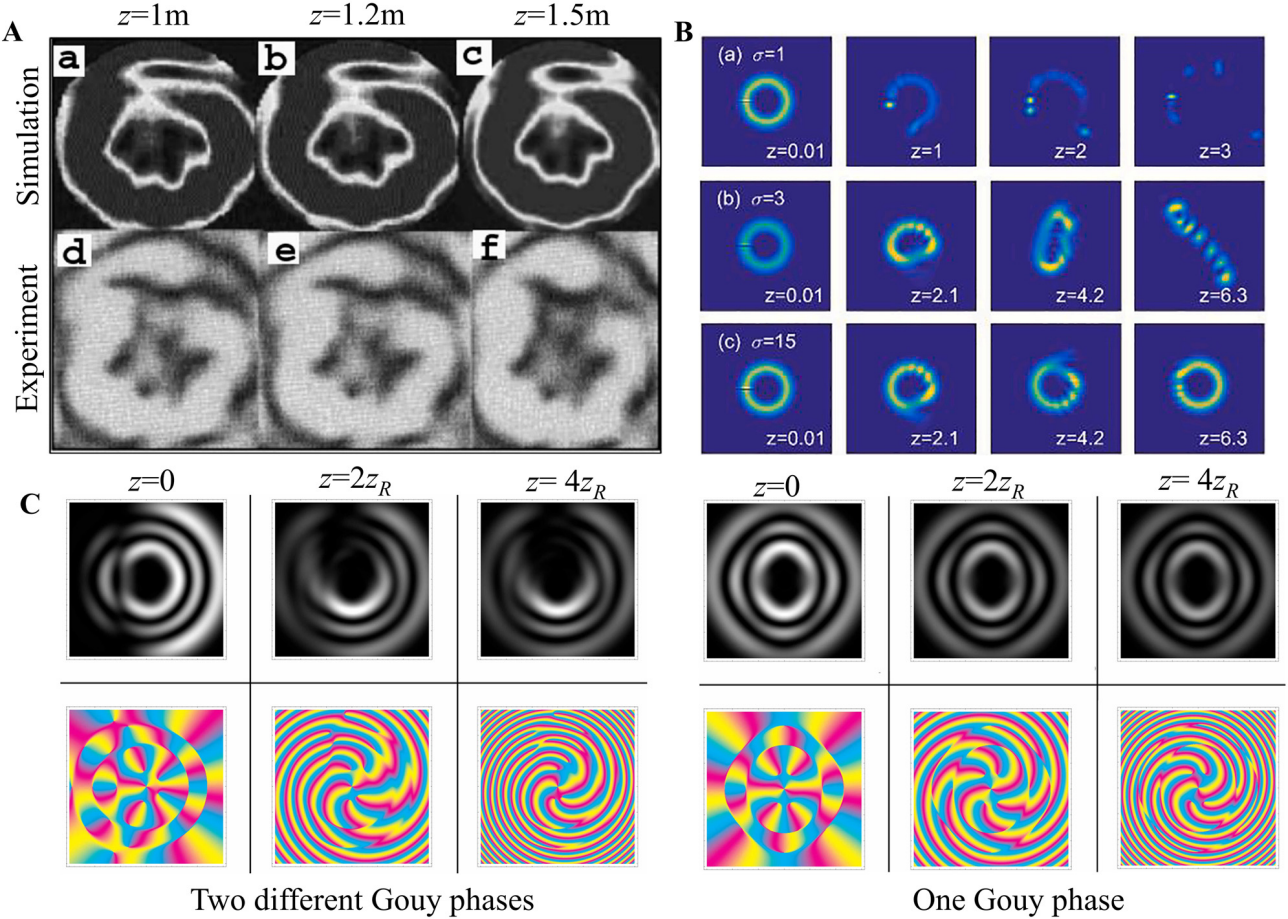
Stability of fractional vortex beam propagation. (A) The diffraction-free property of fractional BG beam. Reprinted from Ref. [84]. (B) Increasing the nonlocality (represented by parameters *σ*). Reprinted from Ref. [88]. (C) Limiting the number of different Gouy phases in the superposition. Reprinted from Ref. [55].

It is widely accepted that fractional vortex beams cannot stably propagate in free space; in 1994, Beijersbergen et al. investigated the fractional LG modes based on a spiral phase plate (SPP) and showed that a single-phase singularity of a higher-order fractional vortex beam splits into several unit phase singularities during propagation in free space [41]. In 1998, Vasnetsov et al. found that a beam with a mixed screw-edge dislocation (referred to as a fractional Gaussian vortex beam) did not propagate as a self-similar stable object, but transformed into several pairs in the far field [43]. In fact, Franke et al. emphasized that a fractional LG beam can be considered as sums of LG modes with different integer TCs that are unstable and do not maintain their amplitude distribution upon propagation because of the different Gouy phases possessed by each integer mode that can be attributed to unstable propagation [103]. Later, Berry considered a simple physical treatment of optical processes in a complex beam field and predicted a complex-phase structure comprising many vortices at various positions for the propagation of the fractional vortex beam [45] that stimulated a torrent of publications on the problem [47, 79, 89, 104, 105]. Subsequently, schemes to improve the transmission stability of fractional vortex beams have been proposed [49, 55, 88, 98, 105]. In contrast to the fractional Gaussian vortex beam, a fractional BG beam is formed due to the addition of a fractional vortex phase to the diffraction-free BG beam. It is still diffraction-free for a working distance, while the central spot and fractional helical wavefront are maintained, and it was also proven to be able to overcome a block of obstacles and regenerate itself after a characteristic distance (see Figure 4A) [49, 84]. In particular, Gotte et al. used this flexibility to determine a representation of a fractional OAM state in terms of LG beams with a minimal number of different Gouy phases, to increase propagational stability (see Figure 4C) [55]. Fractional vortex beams generated in this manner are an excellent realization of noninteger OAM states, and they are more stable during propagation than when light emerges from fractional phase steps. In addition, the vector properties of a beam [98, 106] and the nonlocal properties of the transmission medium [88] have been proven to stabilize such a beam transmission (see Figure 4B).

### 3.2 Beam shaping and rotation

**Figure 5:**
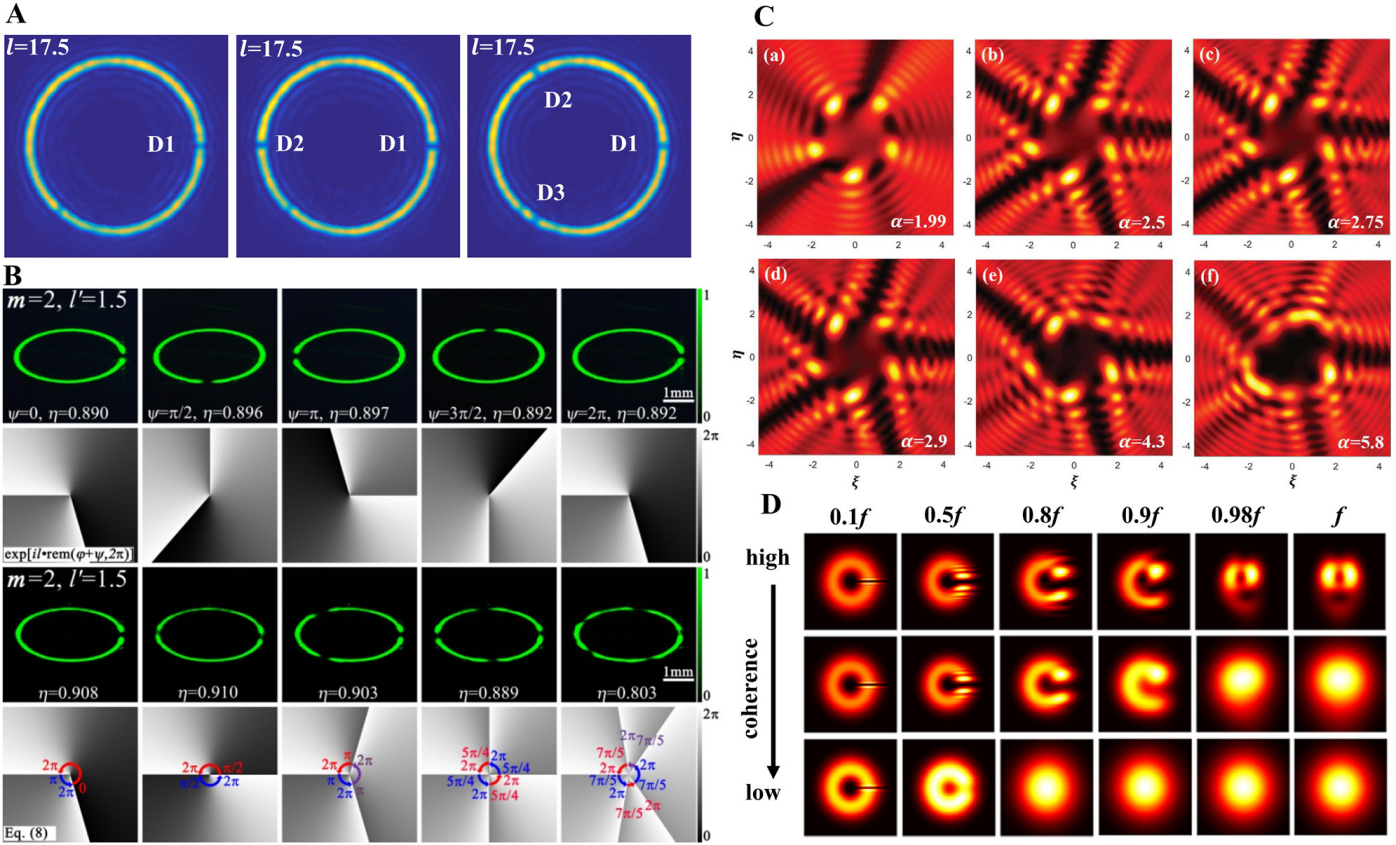
Diversification beam shaping and rotation of fractional vortex beams. (A) Tunability of the number of radial gaps in a perfect fractional vortex beam. Reprinted from Ref. [57]. (B) Tunability of the number and direction of radial gaps for a perfect fractional elliptic vortex beam. Reprinted from Ref. [59]. (C) Anomalous multi-ramp fractional vortex beam with multi-radial gaps. Reprinted from Ref. [83]. (D) Diversity shaping and vanishing of rotation effects due to reduced coherence. Reprinted from Ref. [61].

Compared with the integer vortex beam, whose circular symmetric structure is independent of the TC, the fractional vortex beam has an opening gap that changes for different TC and propagation distances. This unusual property makes it applicable in optical sorting [107] as well as in guiding and transporting particles [63]. It is worth noting that carrying OAM allows a fractional vortex beam to maintain the spot rotation effect on propagation, similar to that of an integer vortex beam [55] (see Figure 2A). In particular, as shown in Figure 5A and B, the number of gaps in a perfect fractional vortex beam can be freely modulated, and by adjusting the scaling factor of the fractional BG beam at the object plane, the perfect fractional vortex mode transformation can be easily controlled from a circle to an ellipse with a high mode purity that enables a wider variety of beam shaping [57, 59, 60]. More interestingly, by modifying the phase term of the fractional vortex beam [59, 60, 83, 95], the resulting beam shows multiple gaps simultaneously for multi-particle trapping, as shown in Figure 5C. In addition, in 2018 and 2020, our group introduced the vortex phase with fractional TC into the partially coherent light field, as shown in Figure 5D, and proposed a scalar and a vector partially coherent fractional vortex beam model, respectively [61, 62]. We realized the joint control of their vortex phase and coherence and revealed novel physical effects, such as more diversity beam shaping and beam rotation disappearance effect.

### 3.3 TC jump

**Figure 6:**
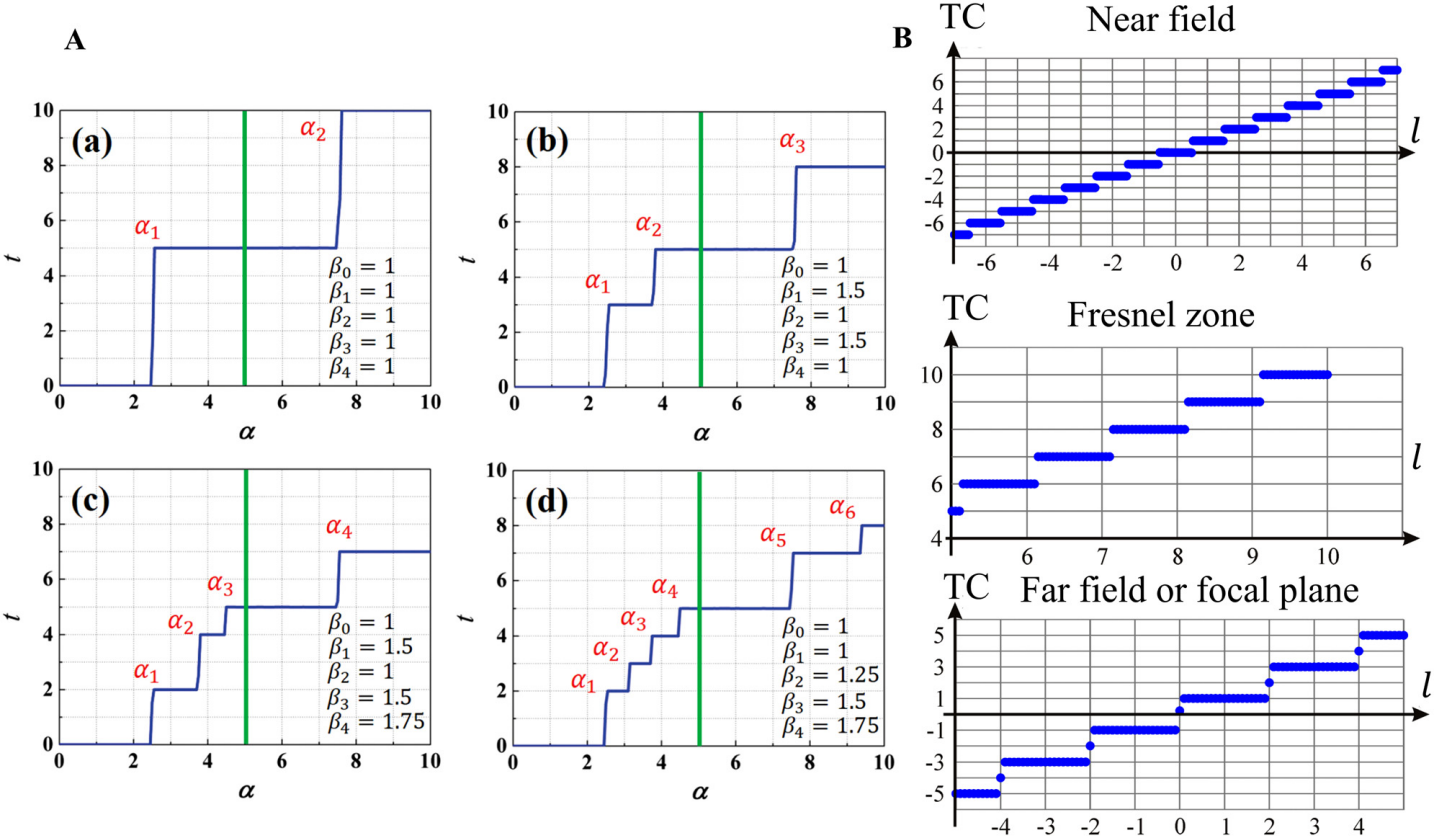
TC jump characteristics of fractional vortex beams. (A) Arbitrary jumps in TC at any critical threshold of the source charge for an anomalous multi-ramp fractional vortex beam. Reprinted from Ref. [83]. (B) Net TC at different propagation planes for a fractional Gaussian vortex beam as a function of the original TC. Reprinted from Ref. [109].

In 2004, Berry showed that no fractional-strength vortices can propagate; instead, they produce a pattern of strength-1 vortex lines, whose total strength is the nearest integer to fractional TC [45]. The propagation wave of a fractional vortex beam is expressed as a superposition of waves with an integer TC. Leach et al. experimentally confirmed that after propagation, fractional vortex beams have intricate phase structures comprising a chain of alternating charge vortices along the direction of the initial radial discontinuity [47]. Furthermore, the visualization process of the formation and evolution of a vortex with an increase in the fractional TC has been reported [79, 80, 89], even at the submicron scale [105]. However, in a demonstration of fractional vortex beams generating new TC through the Hilbert Hotel mechanism, Gbur [46] showed that an adjustable multi-ramp SPP, with *M* (*M* = 1, 2, 3…) ramps in the azimuthal direction instead of one, could cause a jump of *M* in TC as the source charge increased, corresponding to *M* rooms being simultaneously freed in Hilbert’s Hotel. Recently, we further modified the transmission function of the SPPs to design an anomalous multi-ramp SPP that generated an anomalous multi-ramp fractional vortex beam, and demonstrated its rich and varied TC jump characteristics, as shown in Figure 6A [83]. In particular, Gutiérrez-Vega et al. [108] limited the radius of the SPP to produce a fractional vortex beam and found that the pattern of the resulting beam has a spiral-like nature and is finite owing to the effect of the diffraction waves caused by the border SPP that is different from that predicted by Berry. Moreover, the total TC of the fractional vortex beam is always zero because of the limitation of the radius of the SPP. In addition, Jesus-Silva et al. studied the jump characteristics of TCs at the Fraunhofer diffraction distance and far field (focal plane), and the results were also different from those predicted by Berry [81, 89]. Recently, Kotlyar et al. [109] systematically summarized the TC jump characteristics of fractional vortex beams. It is shown that there are four evolution scenarios for an original fractional vortex beam that depend on the position of the observation plane and the proximity of the original TC to an even or odd integer, as shown in Figure 6B.

### 3.4 OAM oscillations

**Figure 7:**
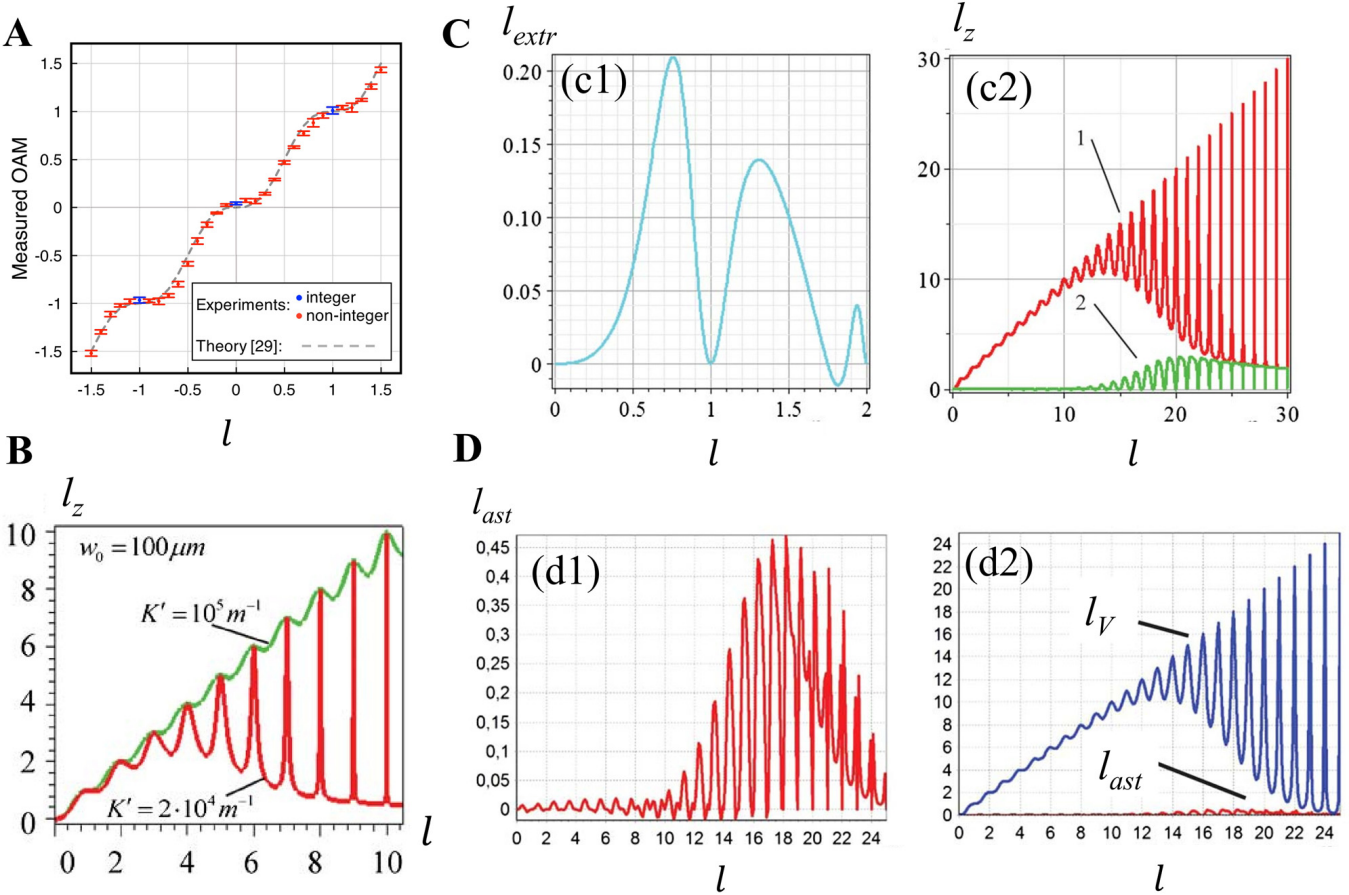
Relationship between the original TC and the total OAM content of the beam. (A) The total OAM presents an oscillatory distribution represented by the expression $l_{z}=l-\text{sin}\left(2l\pi \right)/\left(2\pi \right)$ as the original TC *l* increases. Reprinted from Ref. [110]. (B) The influence of the source parameter on the oscillation distribution of OAM. (C) Contribution of the extrinsic OAM caused by the displacement of the center of gravity to the total OAM. (D) Contribution of the astigmatism OAM caused by the astigmatic transformation of the beam and the optical vortices OAM caused by the vortex to the intrinsic OAM. (B–D) Reprinted from Ref. [111].

The authors remarked that the OAM *l*_z_ follows the fractional vortex TC *l* with small oscillations near the line *l*_*z*_ = *l* for optical vortices with a low-order TC |l<6| , that is, $l_{z}=l-\text{sin}\left(2l\pi \right)/\left(2\pi \right)$ . This was experimentally confirmed in a previous study [110], as shown in Figure 7A. However, a detailed analysis [111] showed that there are OAM pulses with larger amplitudes for higher-order vortices near integer-order values, as shown in Figure 7B. Thus, optical vortices with a fractional-order TC in these studies are associated with the fractional values of OAM. In general, an important property of fractional vortex beams is that their intrinsic OAM per photon can assume any arbitrary value within a continuous range, either integer or noninteger in units of *ħ* [47, 48]. In 2014, Fadeyeva et al. [111] analyzed the oscillation behavior of the OAM in fractional vortex beams for determining the reasons causing such an effect that is connected with the extrinsic and intrinsic OAM (the contribution of the displacement of the center of gravity, the vortex, and astigmatism influence), as shown in Figure 7C and D. Particularly, in 2018, using Fermat’s spiral slit, Yang et al. [112] proposed and experimentally studied an anomalous Bessel vortex beam that carries decreasing OAM along the propagation axis in free space; this is an easy method for modulating the beam TC to be an arbitrary value, both integer and fractional, within a continuous range. Subsequently, Wang et al. extended this structural design that can be used to generate variable TCs, to nanodevices [113].

## 4 Experimental generation of fractional vortex beam

In this section, we discuss the general methods for experimentally generating the aforementioned six types of fractional vortex beams. In general, a fractional vortex beam can be produced based on an SPP with a noninteger phase step, computer-generated holograms, spiral slit, and metasurface, among which, the noninteger SPP is the most classical and efficient method for generating a fractional Gaussian vortex beam. However, the SPP requires a precise manufacturing process and has low modulation freedom. In addition, the spiral slit can realize a fractional BG beam with a variable TC, while causing a large waste of energy. As a general method of realizing various types of fractional vortex beams, computed holography is employed via a phase modulator (for example, spatial light modulator (SLM)). However, it can only produce a fractional vortex beam in free space, where the propagation distance is larger than the wavelength and has a higher energy loss. Until recently, a light beam with a helical wavefront and carrying OAM was produced by transforming the spin angular momentum (SAM) into OAM with a carefully designed complex metasurface. The integrated fractional vortex beam at the nanoscale has potential applications in modern communication systems for further boosting the capacity of transmission channels. In addition, a fully coherent or partially coherent vector fractional vortex beam can also be generated by modulating their polarization states, which is not discussed here [61, 95, 97], [98], [99], [100], [101].

### 4.1 Fractional Gaussian vortex beam generation

**Figure 8:**
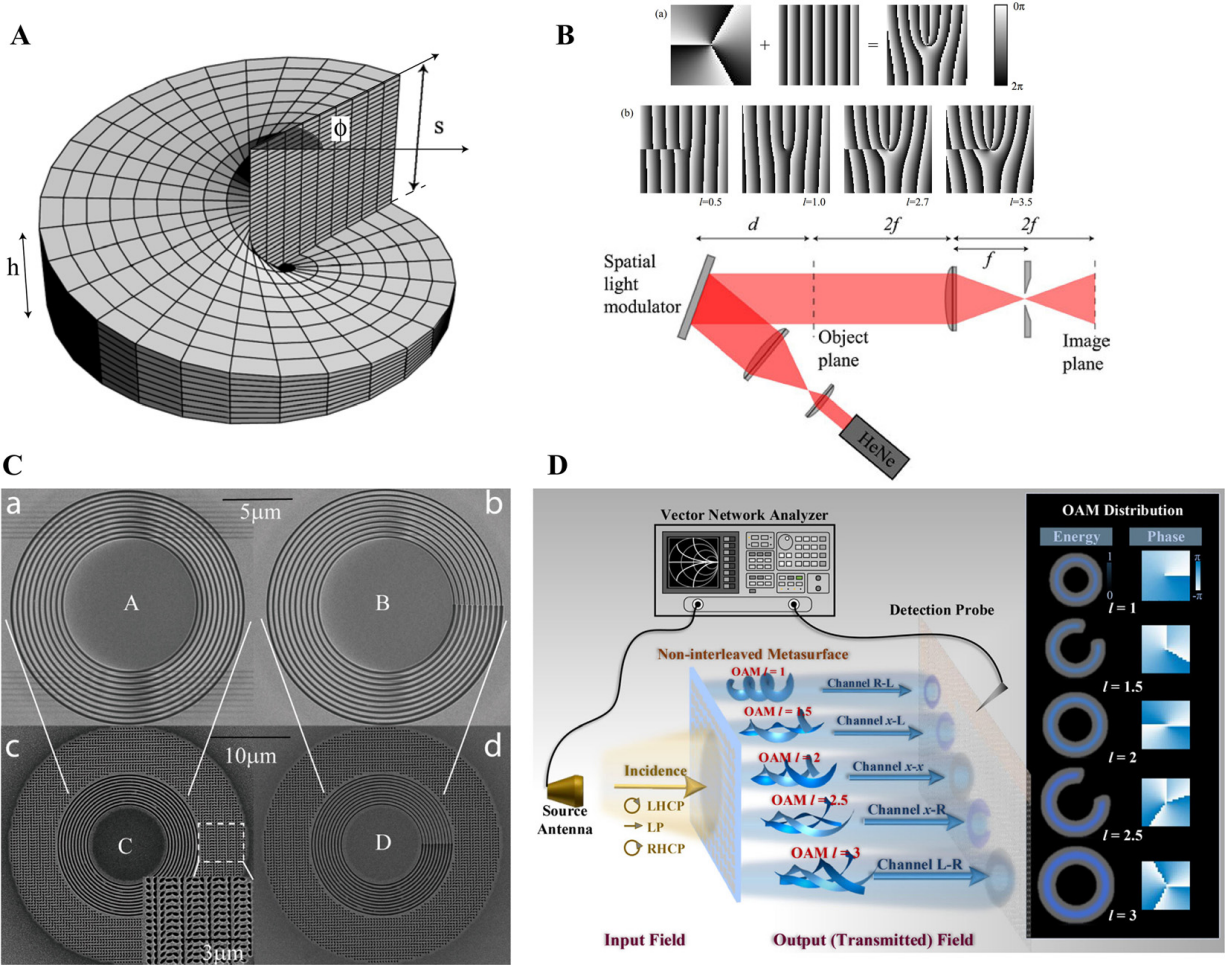
Fractional Gaussian-vortex beam generation via SPP and computer-generated hologram. (A) SPP with phase step 2π*l*. (B) Computer-generated hologram with integer and fractional TCs, and experimental setup for generating a fractional Gaussian vortex beam. (A) and (B) are reprinted from Ref. [47]. (C) Scanning electron microscopy images of annular apertures with an invariant aperture width of 200 nm, and the aperture width increasing from 70 to 280 nm for each ring. Reprinted from Ref. [129]. (D) Schematic principle for metasurface-based versatile generation of integer and fractional Gaussian vortex beam through polarization modulation. The output wavefront with integer and fractional TC *l* = 1, 1.5, 2, 2.5, and 3 are realized by altering the polarization state of the incident wave. Reprinted from Ref. [119].

In general, a fractional Gaussian vortex beam can be generated via a noninteger SPP [41, 105], computer-generated holograms [47, 53, 104, 114], [115], [116], [117], and metasurfaces [118, 119], for imposing a fractional-order spiral phase into the wavefront of the Gaussian beam. The earliest and most general method to produce a fractional Gaussian vortex beam is to pass light through the noninteger SPP [41, 47]. In practice, the SPP is a transparent disc with periodically changed thickness along the azimuthal position, as shown in Figure 8A. Hence, a height step is located at the position *ϕ* = 0, and the desired value of TC *l* has a relationship between step height *s* by *s* = (*n*_*r*_ − 1)*λl*, where *n*_*r*_ is the refractive index of the SPP, and *λ* is the wavelength. However, the precise fabrication of an SPP is necessary to obtain the desired TC for a definite wavelength, while it is difficult to realize a high-order fractional vortex beam that limits its applications in the optical field.

To address this problem, a simple and flexible method (computed holography) based on the phase-only diffractive optical component SLM is proposed to produce a fractional Gaussian vortex beam [47]. The forked computer-generated holograms with integer and fractional TC and the corresponding experimental setup are shown in Figure 8B. Computer-generated holograms are generated via interference between a spiral phase and a blazed grating phase that has a number of |*l*| pronged fork dislocations along the beam axis. Here, a linearly polarized He–Ne laser beam is expanded by two lenses that illuminate the SLM, where the fractional-order fork grating is loaded by the computer. Subsequently, the output modulated light is focused by a lens, and the fractional Gaussian vortex beam in the first order of the diffracted beam is filtered. Furthermore, based on the computed holography method, the fractional vortex lens [120], Fresnel zone plates [121, 122], and second-harmonic generation with two fork grating holograms [123, 124] have been proposed to generate a fractional Gaussian vortex beam. In general, various structures of the fractional Gaussian vortex beam with multiple singularities can also be generated by designing a specific spiral phase [46, 52, 83].

The aforementioned fractional Gaussian vortex beam is macroscopic and is generated in free space. The limitation is that the generation device is cumbersome and is not suitable for nanophotonic systems and integration. To address this limitation, based on the coupling between SAM and OAM, various structured metasurfaces have been proposed to generate light with an integer OAM [125], [126], [127]. The metasurface at the nanoscale process enables faster control of the OAM of the light and can be integrated into a chip. Moreover, the transmission properties of a metasurface can be expressed using a 2 × 2 Jones matrix [128]. Here, the SAM is carried by a light beam with a circular polarization state, and the sign of the OAM is determined by the circular polarization of the incident light (left/right). However, the geometric phase caused by the spin–orbit interaction can only realize an integer OAM. To produce a fractional vortex beam, Guo et al. proposed a continuously shaped metasurface with a controllable plasmonic annular aperture width, as shown in Figure 8C [129]. The proposed metasurface can simultaneously induce the geometric and plasmon retardation phases. When the aperture width is uniform or increases from 70 to 280 nm, the plasmon retardation phase will have an integer multiple change of 2π or π, respectively. By merging these two phases, a beam with an arbitrary TC (integral and fractional) can be produced. Furthermore, Yang et al. proposed a metasurface that was designed based on a single-layer broadband meta-atom with a deformed square loop structure that was verified to generate a fractional vortex beam within a wideband range of 8.55–19.95 GHz and is highly efficient [130]. To further enhance the information capacity of metasurfaces, as shown in Figure 8D, Zhang et al. proposed a paradigm-shifting perspective of noninterleaved metasurfaces with five metallic layers and four substrate layers [119] that occupied the co- and cross-polarization channels and modulated both the geometric and propagation phases. As a result, different integer and fractional Gaussian vortex beams can be generated by adjusting the polarization states of the incident light that illuminates the specific matasurface. In addition, various methods have been employed to generate fractional vortex beams, such as nonlinear wave mixing [131], Dammann vortex grating [132], bilaterally symmetric grating with an aperture metadevice [133], conical diffraction in biaxial crystals [134, 135], and tunable vortex microlasers [136].

### 4.2 Fractional Bessel–Gaussian beam generation

**Figure 9:**
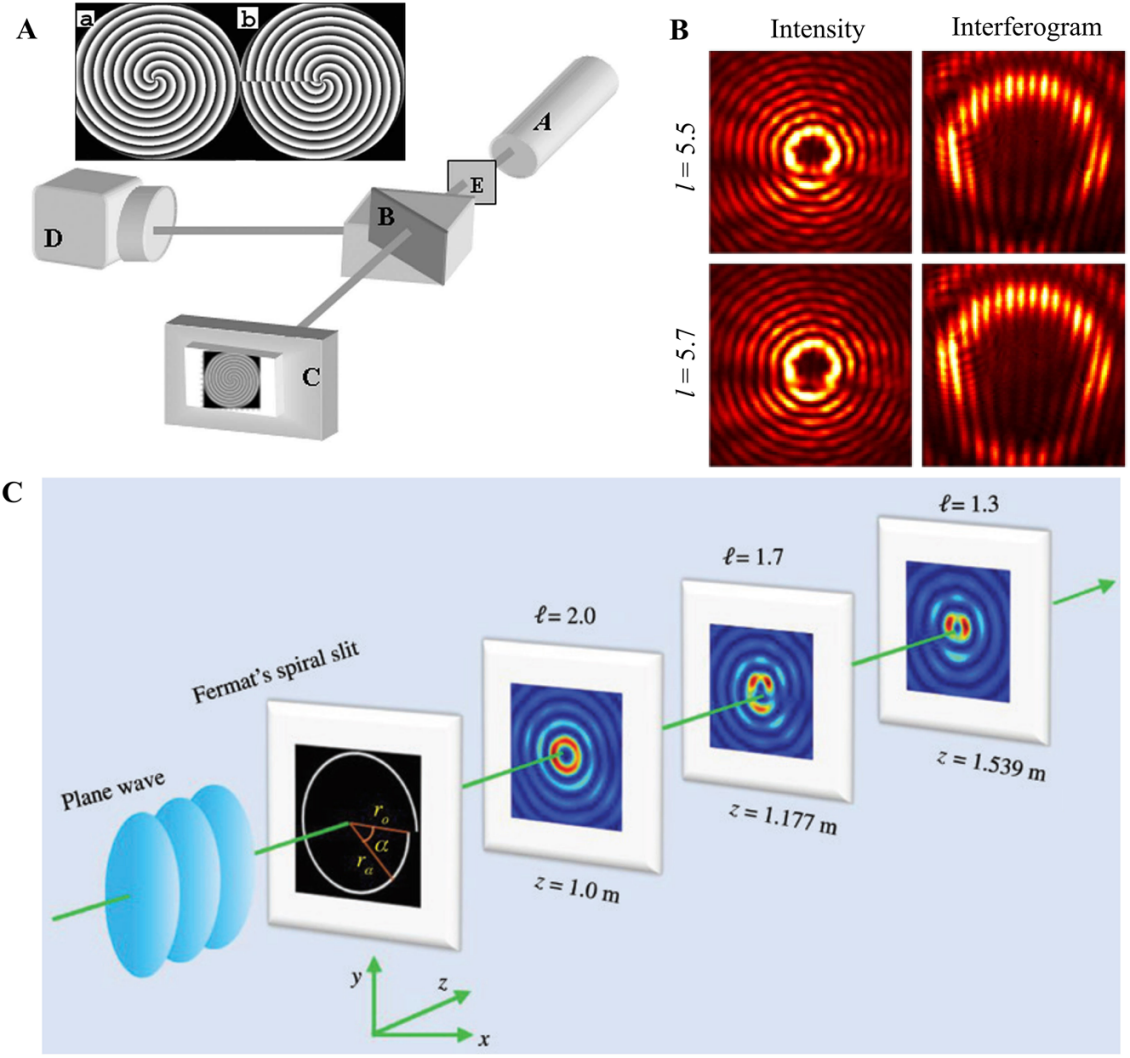
Experiment generation of fractional BG beam. (A) Experimental setup of the fractional BG beam, and the computer-generated hologram with TC *l* = 4 and 4.5. Reprinted from Ref. [84]. (B) The experimental intensity and interferogram patterns of the fractional BG beam with TC *l* = 5.5 and 5.7, respectively. Reprinted from Ref. [86]. (C) Schematic of the generation of the fractional BG beam based on Fermat’s spiral slit. Reprinted from Ref. [112].

Unlike the fractional Gaussian vortex beam, the fractional BG beam is an approximate nondiffracted beam and has multiple light rings. Here, we introduce two classical methods for producing a fractional BG beam experimentally. First, the integer BG can be generated via a vortex beam transmitted through an axicon [137]. Furthermore, computed holography allows us to encode the axicon function into an SLM to realize a flexible modulation of the light wavefront [84]. Here, we introduce a flexible method based on computed holography, where computer-generated holograms of integer and fractional BG beams are shown in Figure 9A. The experimental setup is similar to that shown in Figure 8B, where the expanded beam illuminated the SLM, and the modulated light is the desired integer and fractional BG beam. The corresponding intensity patterns of the fractional BG beam with different fractional orders of TC are shown in Figure 9B.

Furthermore, the zeroth-order BG beam can be realized experimentally via the Fourier transform of an annular slit [138]. When the annular slit is changed into a spiral slit, a continuous phase shift is introduced, where a phase singularity is formed. This method can also be used to produce fractional plasmonic vortices [139, 140]. Interestingly, as shown in Figure 9C, a fractional BG beam is generated via a plane wave transmitted into a Fermat spiral slit [112]. The transmitted light in different positions undergo different optical paths and form a vortex beam, where the TC *l* changes from an integer to a fractional order with an increase in the propagation distance *z*. Finally, the fractional BG beam can be realized using aluminum metasurfaces, where the phase structure is designed based on caustic theory [141].

### 4.3 Fractional Laguerre–Gaussian beam generation

**Figure 10:**
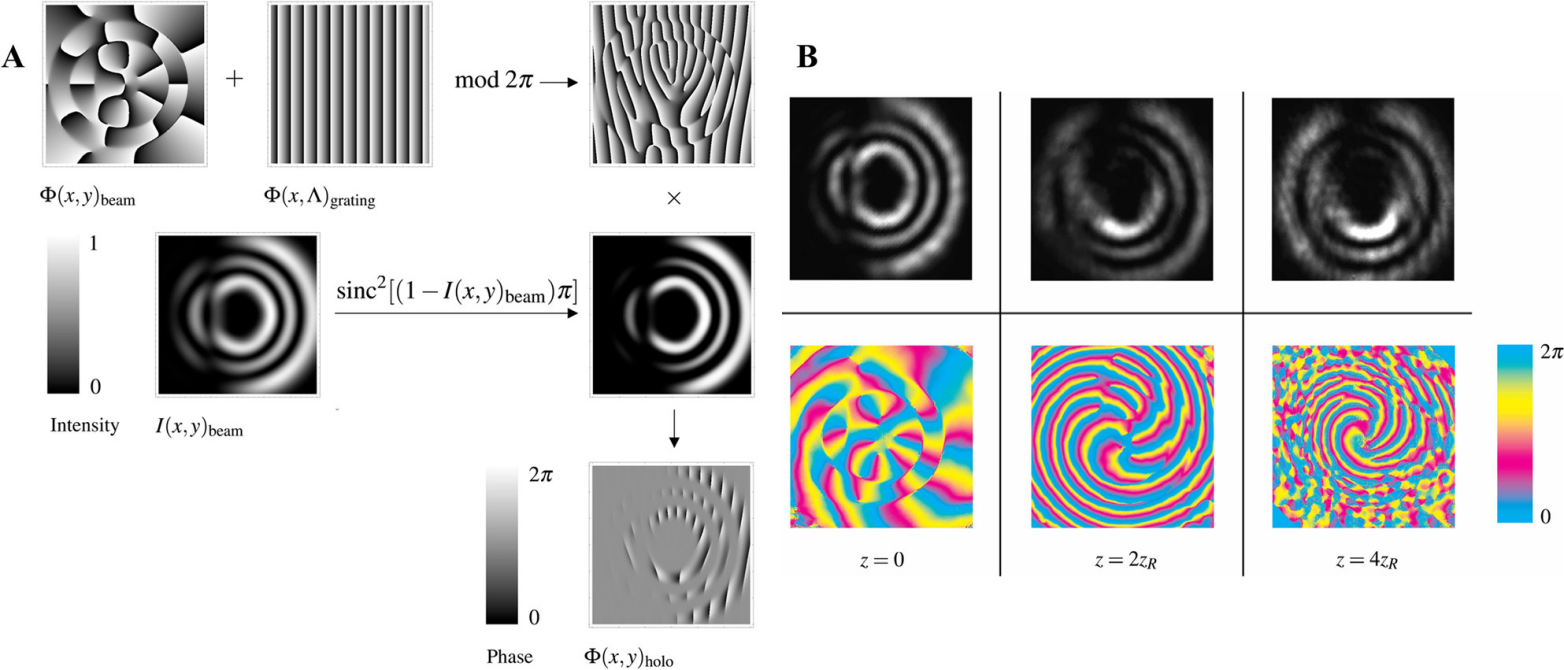
Experimental generation of fractional LG beam. (A) Generation process of the computer-generated hologram via the method of amplitude and phase modulation. (B) Experiment intensity and phase of the fractional LG beam with TC *l* = 6.5 at three planes with different propagation distances (*z* = 0, 2*z*_R_, and 4*z*_R_). Reprinted from Ref. [55].

In contrast to the aforementioned fractional vortex that requires only phase modulation, the fractional LG beam requires both phase and amplitude modulation. The general method to produce a fractional LG beam is via a phase-only SLM, with holograms generated by the superposition of the phase and amplitude structures. The transmission function of the combined holograms can be written as [142]

(18) $$\Phi {\left(x,y\right)}_{\text{holo}}=\left[\left(\Phi {\left(x,y\right)}_{\text{beam}}+\Phi {\left(x,\Lambda \right)}_{\text{grating}}\right)\text{mod }2\pi -\pi \right]{\text{sinc}}^{2}\left[\left(1-I{\left(x,y\right)}_{\text{beam}}\right)\pi \right]+\pi \text{,}$$

where Ф(*x*, *y*)_beam_ and Ф(*x*, Λ)_grating_ are the phases of the fractional LG beam and blazed grating, respectively. Furthermore, Λ denotes the period of grating. Based on Eq. (18), the amplitude-modulated phase computer-generated hologram is generated, as shown in Figure 10A. The generation process is similar to a fractional Gaussian vortex beam with an SLM, except for the hologram design. It should be noted that the phase of the fractional LG beam can be generated via mode superposition or a noninteger spiral phase [55]. Figure 10B shows the corresponding intensity and phase patterns of the fractional LG beam at different propagation distances. Here, the phase of the beam encoded in the hologram is generated via mode superposition, where the fractional LG beam retains an appropriate propagation property. In addition, various fractional LG beams can be experimentally generated via this method by designing a specific fractional spiral phase of a beam.

### 4.4 Perfect fractional vortex beam generation

**Figure 11:**
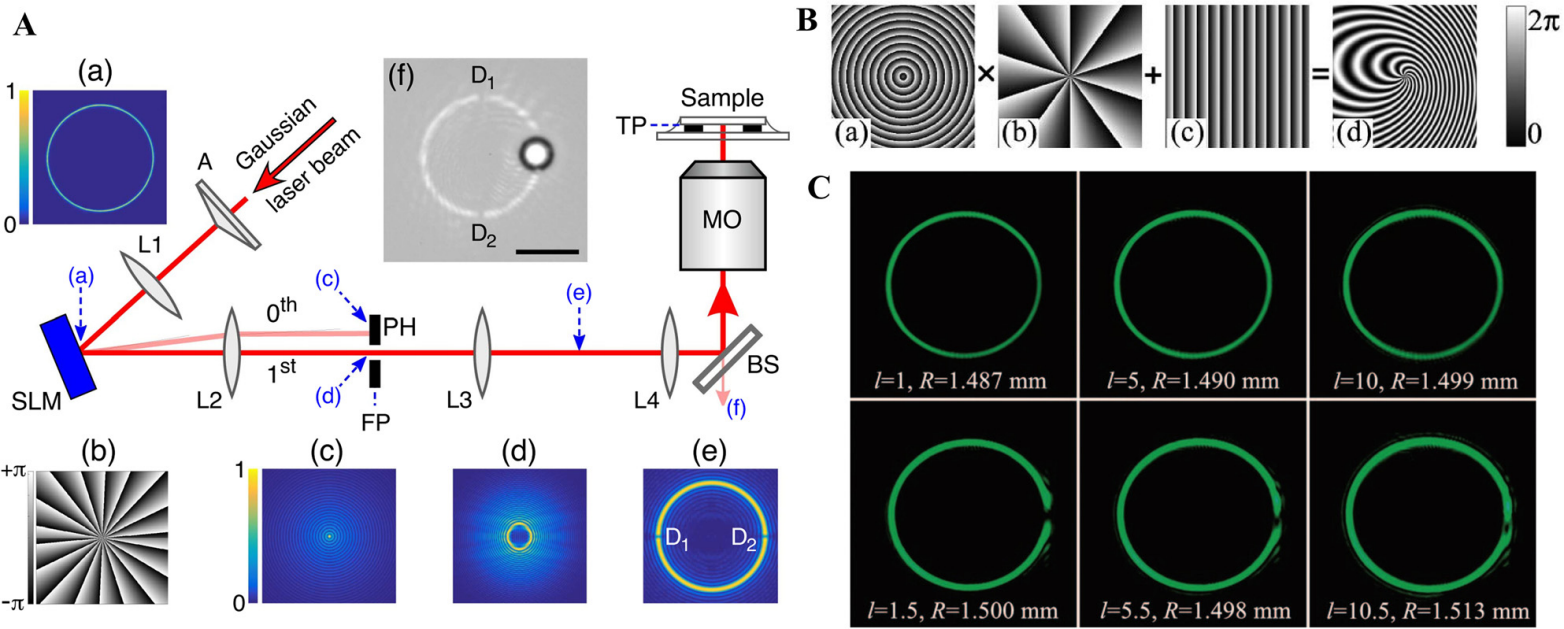
Experimental generation of perfect fractional vortex beam. (A) Schematic and intensity patterns of perfect fractional vortex beam generation by axicon and computer-generated hologram with fractional spiral phase. Reprinted from Ref. [57]. (B) The process of encoding the axicon phase into the computer-generated hologram. (C) Intensity distributions of the integer and perfect fractional vortex beam. (B) and (C) are reprinted from Ref. [145].

The ideal perfect vortex beam possesses a complex amplitude *δ*(*r* − *r*_0_)exp(i*lθ*) that cannot be produced in the experiment. Approximately, the perfect vortex beam can be generated by the Fourier transform of the higher-order BG beam [143, 144] that led the researchers to believe that the perfect fractional vortex beam can also be generated by introducing a fractional-order TC to the BG beam. As shown in Figure 11A, a ring-shaped light is generated via a collimated laser beam transmitted through axicon A and lens L_1_. Subsequently, the ring-shaped light illuminates the SLM that is used to encode the fractional spiral phase. After Fourier transformation, a perfect fractional vortex beam is produced in the Fourier plane of lens L_2_. The experimental setup consisted of a 4-*f* system to relay a perfect fractional vortex beam into the back focal plane of the microscope objective for optical trapping [57]. Furthermore, the axicon can also be replaced by encoding the axicon transmission function exp[−i*k*(*n*_*r*_ − 1)*ra*] into the computer-generated hologram of the SLM, as shown in Figure 11B. Here, *k* is the wave number, *n*_*r*_ is the refractive index, and *a* is the cone angle of the axicon [145]. From the experimental results shown in Figure 11C, the perfect fractional vortex beam has a gap in the light ring and maintains the same radius with varying TC.

### 4.5 Fractional elliptic vortex beam generation

**Figure 12:**
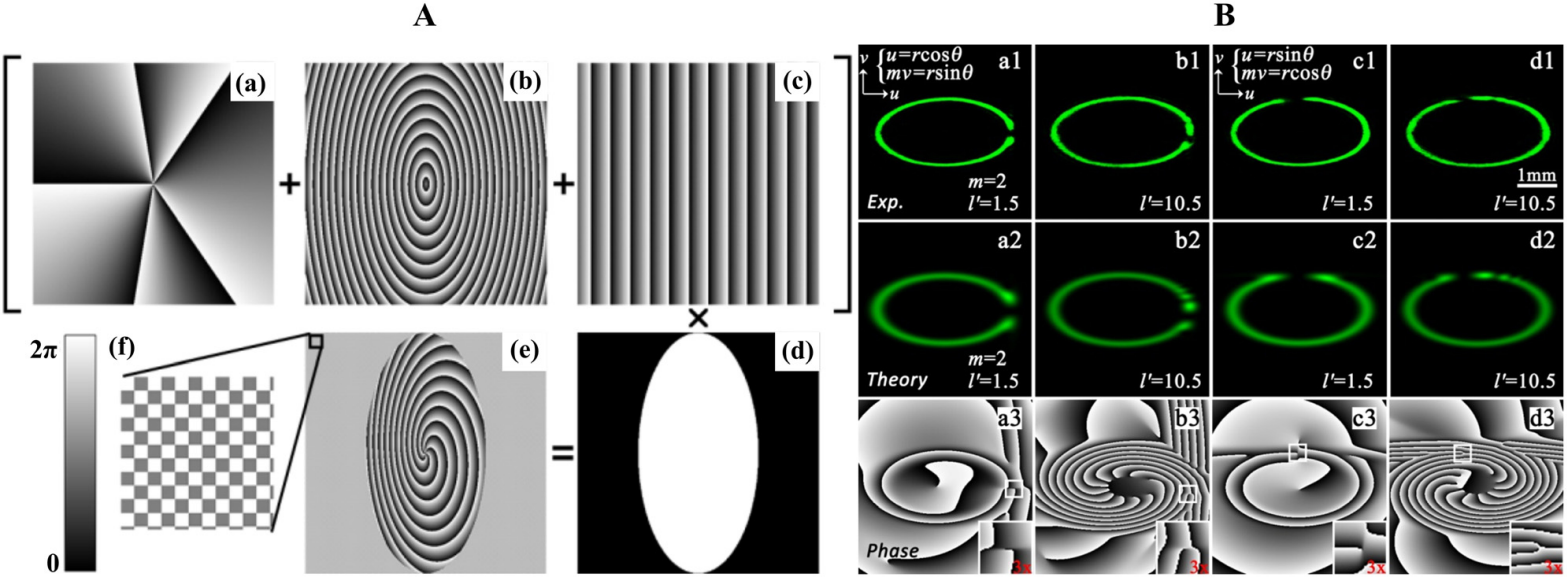
Experimental generation of a fractional elliptic vortex beam. (A) Generation process of the computer-generated hologram of the fractional elliptic vortex beam. (B) Experimental intensity patterns and theoretical intensity and phase patterns of the fractional elliptic vortex beam. (A) and (B) are reprinted from Ref. [59].

In addition to the circular structure, the fractional vortex can also achieve an elliptical structure based on the coordinate transformation method that is discussed in Section 2.1.5 [59]. In the experiment, the fractional elliptic vortex beam can be generated via SLM, where the encoded computer-generated holograms are obtained based on elliptic coordinates. As an example, the computer-generated hologram generation process of the perfect fractional elliptic vortex that plays a crucial role, is depicted in Figure 12A. The hologram is generated by combining the vortex, axicon, blazed granting phases, and an elliptic aperture. If the axicon phase is deleted, the perfect fractional elliptic vortex beam will be transformed into a fractional elliptic Gaussian vortex beam. As a result, different perfect fractional elliptic vortex beams with controllable gap positions are generated by modulating the elliptic coordinates, as shown in Figure 12B. Furthermore, fractional elliptic vortex beams with other types can also be generated by modulating the vortex phase and amplitude via the coordinate transformation method [60, 146].

### 4.6 Partially coherent fractional vortex beam generation

**Figure 13:**
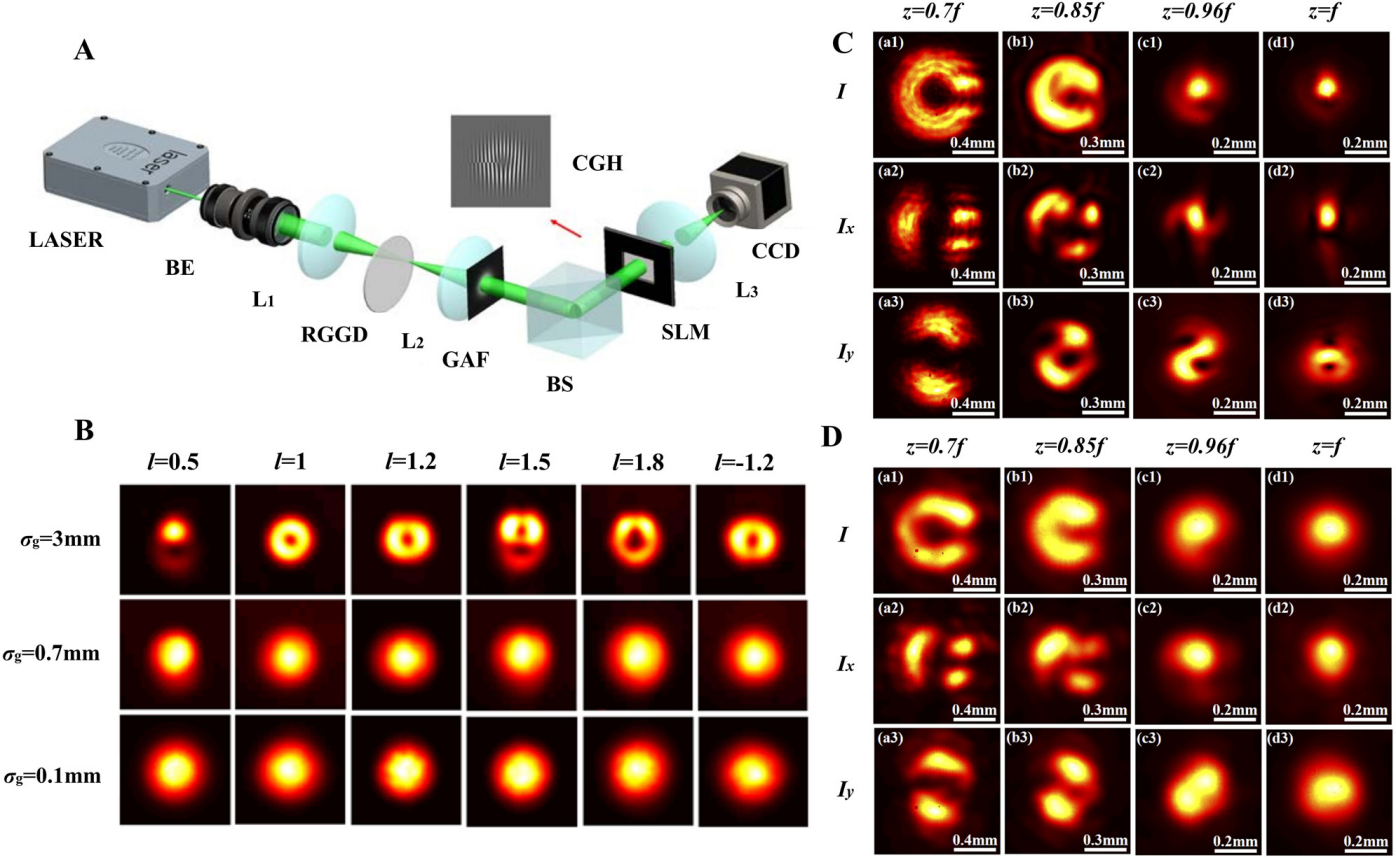
Generation of the partially coherent fractional vortex beam. (A) Experimental setup for generating PCFV beam and measuring the focused intensity distribution and the modulus of the CSD distribution. Laser, Nd: YAG laser; BE, beam expander; L1, L2, and L3, thin lenses; RGGD, rotating ground-glass disk; GAF, Gaussian amplitude filter; BS, beam splitter; SLM, spatial light modulator; CGH, computer-generated holograms; CCD, charge-coupled device. (B) Experimental normalized intensity patterns with different TCs and different coherence widths. (A) and (B) are reprinted from Ref. [61]. (C) and (D) Experimental normalized intensity *I* and its component *I*_*x*_ and *I*_*y*_ of the partially coherent radial polarization fractional vortex beam with fractional TC *l* = 1.5, beam waist *w*_0_ = 1 mm and coherence width *σ*_g_ = 3 and 0.8 mm, respectively. (C) and (D) are reprinted from Ref. [62].

A partially coherent fractional vortex beam is generated in two steps. The first step is the generation of a partially coherent beam, and the second step is the imposition of a fractional spiral phase in the produced partially coherent beam. Figure 13A shows the experimental setup used to generate a scalar partially coherent fractional vortex beam. The laser beam with a wavefront *λ* = 532 nm is expanded by the beam expander and subsequently focused by the thin lens L_1_. The partially coherent beam with Gaussian correlation is generated via the focused beam illuminating on a rotating ground-glass disk, thin lens L_2_, and Gaussian amplitude filter. Next, the partially coherent vortex beam is generated by incorporating the fractional spiral phase into a partially coherent beam with the aid of the SLM. The value of the TC is determined by computer-generated holograms written into the SLM, and the coherence width is controlled by the focused beam spot on the rotating ground glass disk. Figure 13B shows the intensity distributions of the generated partially coherent fractional vortex beam that has a similar intensity (high coherence width) as that of the fully coherent fractional vortex beam and the spot formed with a low coherence width [61]. Furthermore, it is worth noting that a partially coherent fractional vortex with radial polarization states can be generated by adding a radial polarization converter between the rotating ground glass disk and the SLM, as shown in Figure 13A [62]. The corresponding intensity distributions with high and low coherent widths (*σ*_g_ = 3 and 0.8 mm) are shown in Figure 13C and D, respectively.

## 5 Measurement of fractional vortex beam and OAM spectrum

Given the broad applications of vortex beams, various techniques have been proposed for the measurement of TC. For example, the number and direction of forks in the interference patterns of vortex beams with plane waves indicate the magnitude and sign of TC, respectively [113]. The vortex beam can be transformed into a nonhollow spot through the phase grating of the opposite TC that also facilitates the identification of the value of TC [114]. In addition, wavefront measurement is an intuitive technique for obtaining the value of TC, that is, the phase integral around the singularity divided by 2π corresponds to the value of TC [112]. However, the fractional vortex beam breaks the orthogonality of OAM, and its measurement should be modeled as a complicated mixed-OAM case; thus, the conventional methods effective for integer vortex beams become invalid [147].

To qualitatively distinguish the approximate TC of the fractional vortex beam, the diffraction or interference patterns can still be used to identify the fractional TC by comparing the intensity shape with the theoretical ones. Combined with machine learning, a more precise identification can be obtained based on the intensity feature analysis. For further quantitative purposes, that is, high-dimensional optical communication, the TC or OAM spectrum of a fractional vortex beam must be detected. With the development of measurement technology, the results obtained based on modified Mach–Zehnder interferometers [148], [149], [150], [151], [152], mode interconversion [153], [154], [155], [156], dynamic annual double slit [157] and machine learning [158, 159] are increasingly precise. Because the fractional vortex beam can be extended as a Fourier series of integer vortex beams (see Section 2.1.1 and Eqs. (2) and (3)), the measurement methods of the OAM spectrum are also effective for the detection of fractional vortex beams [147, 160], [161], [162], [163].

### 5.1 Measurement of the fractional TC

#### 5.1.1 Modified Mach–Zehnder interferometers

**Figure 14:**
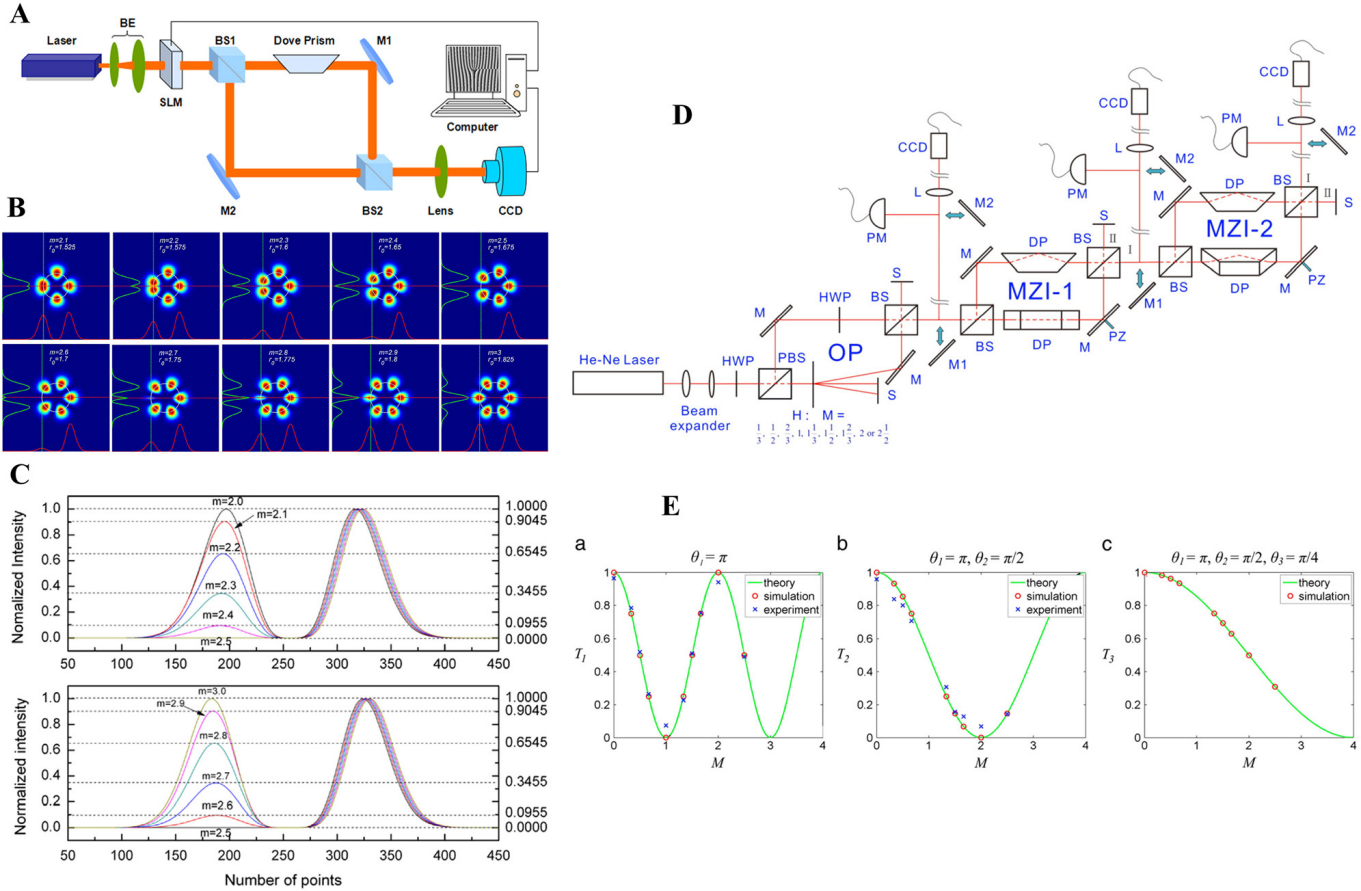
Measurement of the TC of fractional vortex beam based on MZI. (A) Experimental setup to identify the TCs of the fractional vortex beam. (B) Numerical simulations of interference patterns obtained for TC from 2.1 to 3 by steps of 0.1. (C) Intensity curves on the symmetric axes of the interference patterns. (A)–(C) are reprinted from Ref. [148]. (D) Experimental setup with multiple MZIs. (E) The transmittance versus TC in the first three stages. (D) and (E) are reprinted from Ref. [149].

Dove prism can be used to flip images. When a Dove prism is used in a Mach–Zehnder interferometer (MZI), one can easily calculate the magnitude of TC for an integer vortex beam, that is |*l*| = *N*/2, where *N* is the number of ‘petals’ in the flower-like interference pattern (see Figure 14A). Similarly, the interference pattern can also be used to qualitatively identify or quantitatively measure the TC of a fractional vortex beam. The typical interference patterns of the MZI with Dove prism are shown in Figure 14B. In contrast to integer vortices, for a fractional vortex, the intensity of the petals along the symmetric axes of the interference pattern is no longer symmetrical, as shown in Figure 14C. Thus, the ratio of the peak intensity indicates the TC of the fractional vortex beam [148, 149]. In fact, this method is based on the interference patterns between the vortex beam with its conjugate beam, so it cannot be used to determine the sign of the TC.

With multiple MZIs, as shown in Figure 14D, one can obtain a more quantitative calculation of fractional TC by measuring the transmittance of light for each stage, that is, the ratio of the power exiting the interferometer to the power entering it [149]. The transmittance *T*_*M*_ on the *M*_*th*_ (*M* = 1, 2, 3…) MZI stage can be derived as a function of TC and the relative angle $\theta_{M}^{\prime }$ between the Dove prisms. For different stages, the one-to-one relationship between *T*_*M*_ and *l* is progressive (see Figure 14E). With *M* stages used and $\theta_{M}^{\prime }$ for each stage fixed, one can obtain the value of the TC up to $l=2^{M-1}$ by measuring *T*_*M*_ of all stages.

Furthermore, by mimicking the Faraday rotation, the modified MZI is also used to sort the OAM based on the OAM-to-polarization coupling effect [150, 151]. The polarization rotation angle $\varphi^{\prime }$ can be expressed as $\varphi^{\prime }=l\theta^{\prime }$ , where $\theta^{\prime }$ is the relative orientation of the two Dove prisms. A mixture of multiple OAM can be sorted into individual even and odd subsets with θ′ = 90° [152]. This method has the potential for use in the measurement of fractional TC.

#### 5.1.2 Interconversion between Hermite–Gaussian and Laguerre–Gaussian modes

Beijersbergen et al. conducted pioneering work on the interconversion between Hermite–Gaussian (HG) and LG modes with a π/2 mode converter in 1993 [153]. Courtial and Padgett analyzed the performance of a cylindrical-lens mode converter for transforming HG into LG modes [154]. In 2016, a robust setup with a π/2 mode converter was proposed to realize high-order OAM mode conversion up to TC = 100 and it displayed the interesting dynamics of fractional OAM through the converter [155].

**Figure 15:**
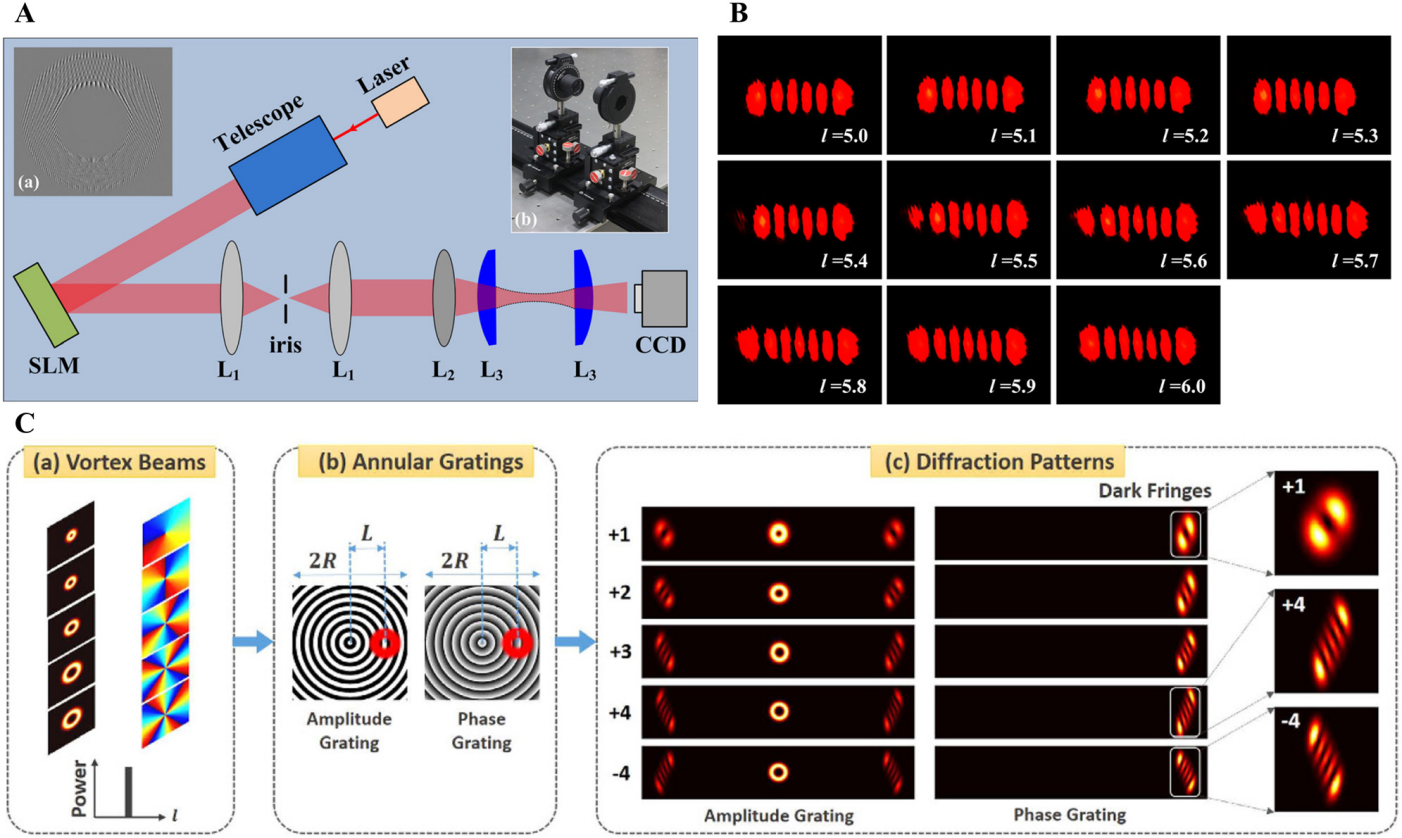
Measure the TC based on HG and LG modes interconversion. (A) An example of holographic grating used for generation of LG_50,5_. (B) Experimental results for fractional vortices with TCs from 5.0 to 6.0. (A) and (B) are reprinted from Ref. [155]. (C) Concept and principle of measuring OAM states of vortex beams with annular gratings. Reprinted from Ref. [156].

Any LG_*pl*_ mode can be expressed as the sum of the HG_*cd*_ modes [153]

(19) $$LG_{pl}=\sum_{M=0}^{N}i^{M}b\left(p,l,M\right)HG_{N-M,M}\text{,}$$

where N=|l|+2p and i^*M*^ corresponds to a π/2 relative phase difference between successive components. As shown in Figure 15A, the holographic grating is designed by adding a blazed grating modulo 2π to a spiral phase term, and subsequently multiplying the phase hologram with the intensity profiles of the LG beams. The $L_{3}-L_{3}$ cylindrical lenses constitute a π/2 mode converter. *L*_2_ is used to modify the output waist of the SLM to satisfy the matching condition. For an integer vortex beam, *p* and *l* can be determined accurately based on *c* and *d*, that is, l=c−d and p=min{c,d} . For a fractional vortex beam, as it can be expressed as a superposition of integer vortex beams, the intensity patterns on the CCD camera correspond to the superposition of a series of HG modes. Thus, an extremely interesting evolution between adjacent integer TC appears, and accordingly, the fractional TC can be identified based on the intensity evolution (see Figure 15B). Similar diffraction patterns and conclusions can also be realized based on amplitude or phase annual grating, as shown in Figure 15C [156].

#### 5.1.3 Dynamic annual double slit

**Figure 16:**
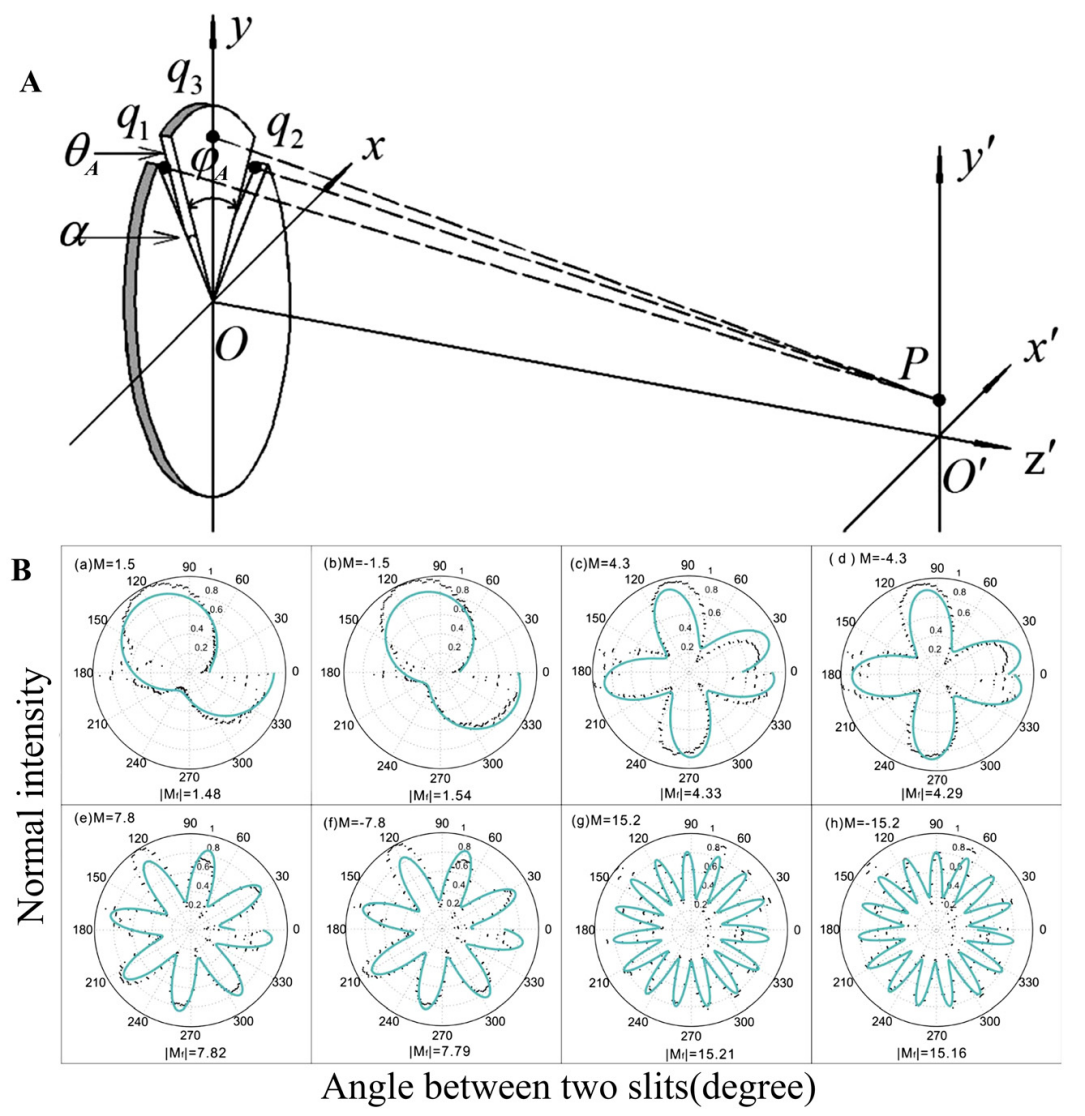
Measurement of the TC based on dynamic annual double slit. (A) Diagram of ADS interference. (B) $I-\varphi_{\text{A}}$ curves in polar coordinates. Here, the additional phase $\theta_{\text{A}}$ is 0, and the black dots and green solid curves are the experimental data and fits to the data, respectively. Reprinted from Ref. [157].

When a fractional vortex beam passes through a dynamic annual double slit (ADS), the interference pattern intensity at *P* (a point on the far field) can be simplified as [157]

(20) $$I\propto 1+\text{cos}\left[i\left(\varphi_{\text{A}}+\theta_{\text{A}}/l\right)\right]\text{,}$$

where $\varphi_{\text{A}}$ is the angle between the double slit of ADS, $\theta_{\text{A}}$ is an additional phase set on one of the slits, and *l* is the fractional TC. As shown in Figure 16A, the angular bisector direction of the dynamic ADS is parallel to the *y* axis and the *y*′ axis. The angular bisector is fixed along the *y* axis and the two single slits are continuously rotated with the same angular velocity with respect to the *y* axis. Thus, a periodic bright or dark intensity can be obtained at the *y*′ axis when a vortex beam illuminates the ADS. After the intensity is collected at *P*, the $I-\varphi_{\text{A}}$ curves can be obtained (see Figure 16B). Subsequently, the magnitude of fractional TC can be obtained by fitting the data using Eq. (20). The sign can be determined by assigning a phase shift $\theta_{\text{A}}$ to one of the slits. If $\theta_{\text{A}}$ is positive, the second curve rotates clockwise when the sign of *l* is positive and anticlockwise, while the sign of *l* is negative. The error of the probing result with this method was less than 5%. In addition, the dynamic ADS method is also suitable for precisely determining the TC of fractional BG beams [164].

#### 5.1.4 Machine learning

**Figure 17:**
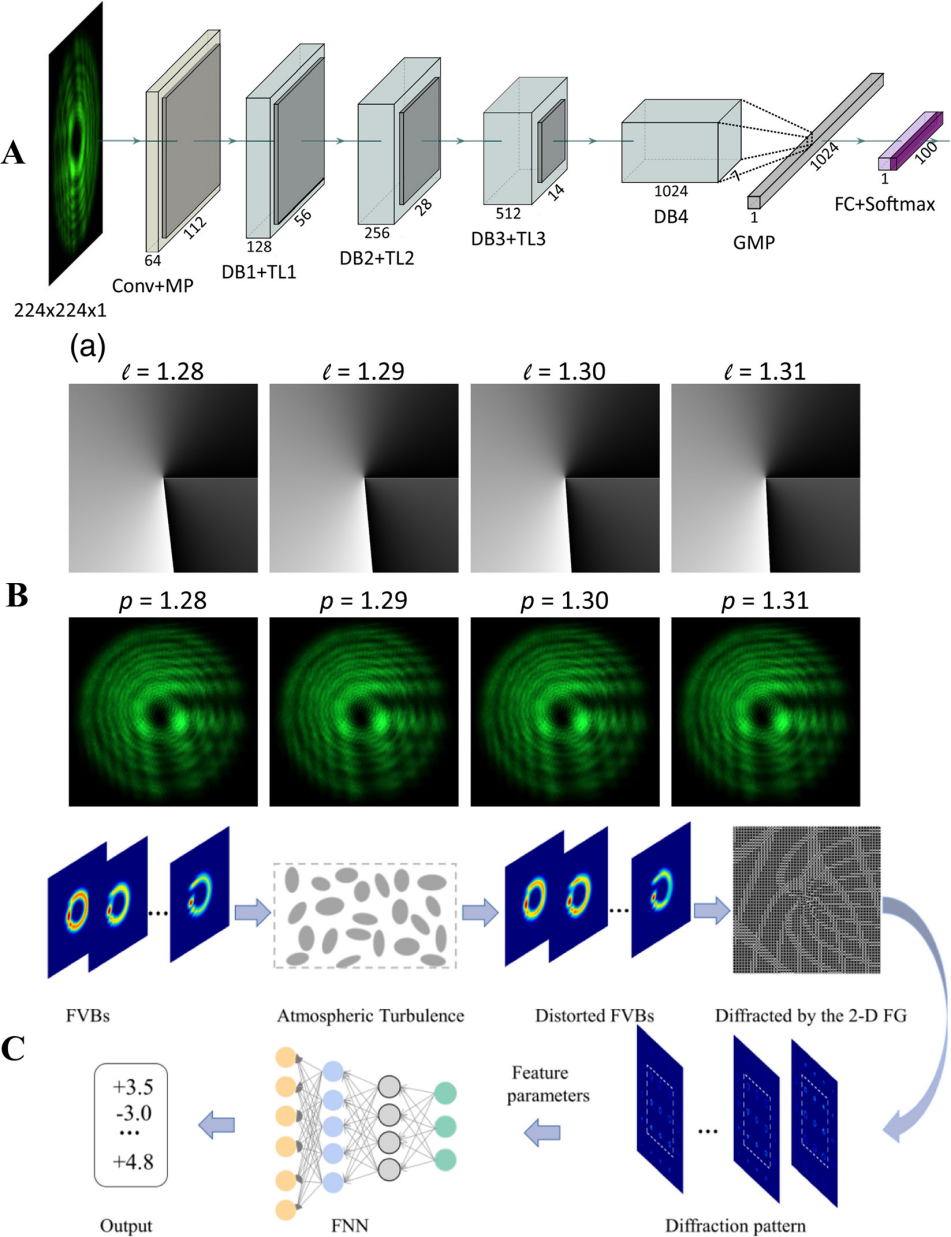
Measurement of the TC by machine learning. (A) Schematic diagram of the OAM-recognition neuron network architecture for recognizing OAM modes. (B) The adjacent fractional OAM modes with 0.01 steps are clearly distinguished. Top row shows the phase patterns uploaded on the SLM and the bottom row shows the recorded intensity patterns. (A) and (B) are reprinted from Ref. [158]. (C) Schematic diagram of the measurement process for the fractional OAM mode. Reprinted from Ref. [159].

The identification of far-field diffraction intensity can also provide a precise determination of the fractional TC. Liu et al. proposed a deep learning method for precisely recognizing OAM modes of fractional vortex beams [158] (see Figure 17A and B). The minimum interval recognized between adjacent modes decreases to 0.01. Regarding disturbances in the turbulence environment, Jing et al. proposed a feedforward neural network where diffraction preprocessing with a two-dimensional fork grating was implemented to endow the feedforward neural network with more feature information [159] (see Figure 17C). The simulation results show that the 9-layer feedforward neural network can identify the fractional OAM mode with an interval of 0.1 and an accuracy of 99.1% under turbulence.

### 5.2 OAM spectrum measurement of the fractional vortex beam

Optical vortices with arbitrary fractional TCs can be written as a Fourier series of integer vortex beams, as expressed in Eqs. (2) and (3). The weight of each integer vortex can be expressed as $C_{n}\left(l\right)=\text{exp}\left(il\pi \right)\text{sin}\left(l\pi \right)/\left[\pi \left(l-n\right)\right]$ . Thereafter, the average OAM of the fractional vortex beam is calculated with $L_{\text{cal}}=\sum_{n=-\infty }^{\infty }n{\left|C_{n}\left(l\right)\right|}^{2}$ . The relation between *L*_cal_ and the fractional TC is *l* = *L*_cal_ + sin(2*l*π)/(2π). As the integer *n* steps away from the fraction *l*, the weight decays to zero, while the weight reaches a peak as *n* equals the closest integer of fraction *l*. Therefore, in the real experiment, *L*_cal_ is determined with $\sum_{n}n{\left|C_{n}\left(l\right)\right|}^{2}$ with a finite number of *n*. Thus, the detection method of fraction *l* will be consistent with the measurement of weight $I_{n}={\left|C_{n}\left(l\right)\right|}^{2}$ of the OAM spectrum. In this section, we review some methods for measuring the OAM spectrum.

#### 5.2.1 Fork grating filter

**Figure 18:**
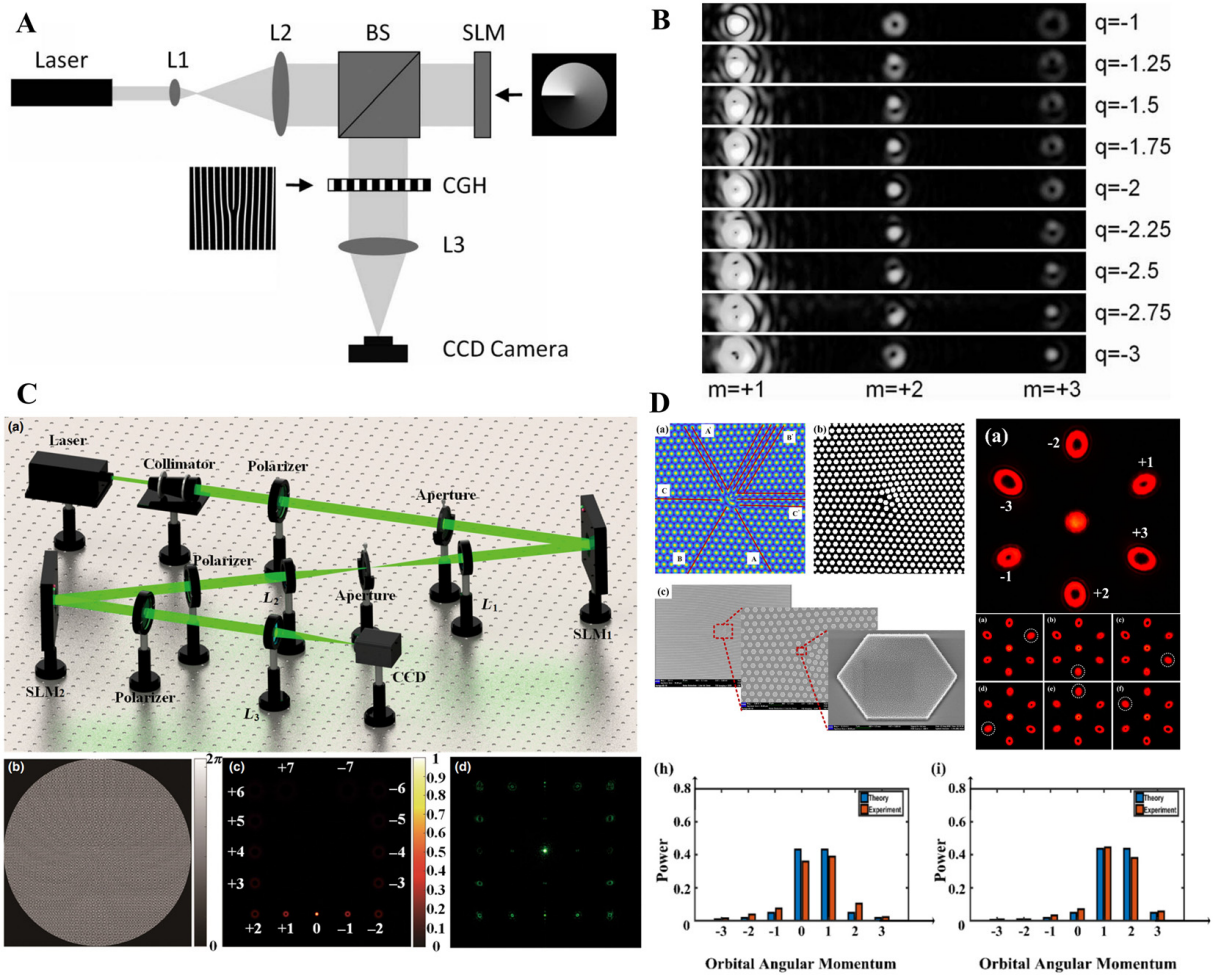
Measurement of the OAM spectrum using the fork grating filter. (A) Experimental setup of the grating vortex spectrum analyzer. (B) Experimental results of the grating vortex spectrum analyzer where the TC of the input vortex ranges from −1 to −3 (marked on the right side of the image). (A) and (B) are reprinted from Ref. [160]. (C) Experimental setup and computer-generated hologram for the generation of the 2D multifocal array. Reprinted from Ref. [147]. (D) An on-chip device for the measurement of the TC of integer and fractional vortex beam. Reprinted from Ref. [161].

As is widely known, the TC of the incident light increases from *l* to *l*′ = *l* + *l*_0_ after passing through a fork grating with TC = *l*_0_. Thus, the contribution of $l=-l_{0}$ can be identified by defining a characteristic point located at the center of the pattern because the *l*′ = 0 term is the only contributor. The rest of the vortex components $l^{\prime }\neq 0$ do not contribute because they all present a null intensity at the center. As shown in Figure 18A, the fractional vortex beam is generated using SLM and illuminates the computer-generated holograms. By applying the computer-generated hologram with adjacent integer TC and subsequently extracting the intensity values in the center of the patterns (see Figure 18B), the value and sign of TC for any input optical vortex can be determined [160].

More efficiently, Duo et al. designed a multifocal array [147], after which an intensity array of different integer components can be measured simultaneously. When the multifocal array is illuminated with a fractional vortex beam, the electric field can be written as

(21) $$E_{\text{DM}}=\left[\sum_{m=m_{1}}^{m_{M}}S_{m}\left(\Delta x,\Delta y\right)\exp\left(im\varphi \right)\right]\sum_{n=\infty }^{\infty }C_{n}\left(l\right)\exp\left(in\varphi \right)\text{,}$$

where *m* = *m*_1_, *m*_2_,… *m*_*M*_ (*M* = 1, 2, 3…) are the integer OAM numbers of the multifocal array, and $S_{m}\left(\Delta x,\Delta y\right)$ corresponds to the focal point position. When *n* equals −*m*, one can see a focal spot at the corresponding focal position (see Figure 18C), and the intensity of the spot will equal the weight of the OAM spectrum. It should be noted that only the intensity of the central point must be measured, and the surrounding ring-shaped intensity generated by the other integer component of the fractional vortex beam should be neglected. By employing a compensation iteration algorithm to correct the detected average OAM of the fractional vortex beam, the error in the average OAM detection will be less than 0.025. Recently, such measurement technology has been incorporated into an on-chip device (see Figure 18D) [161].

#### 5.2.2 Interference

**Figure 19:**
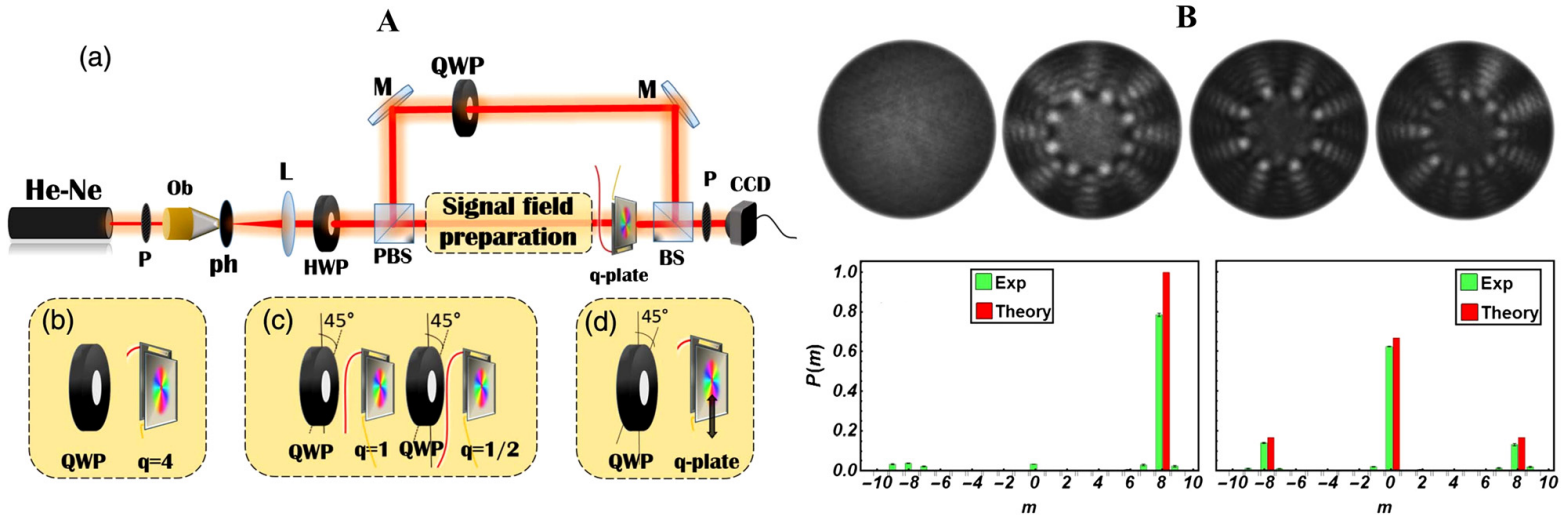
Measurement of the OAM spectrum based on interference. (A) Schematic of the experimental apparatus. (B) Top row: experimental intensities *I*_ref_, *I*_s_, *I*_0_, and *I*_π/2_, respectively. Bottom row: experimental reconstruction of the OAM spectrum. (A) and (B) are reprinted from Ref. [162].

Based on interference theory one can calculate the coefficient of each integer OAM via finite intensity measurement [162]. As shown in Figure 19A, a reference arm is introduced, and the intensity on the CCD plane can be written as

(22) $$I=I_{\text{s}}+I_{\text{ref}}+{\tilde{I}}_{\alpha }\text{,}$$

where *I*_s_, and *I*_ref_ are the signal and reference intensities, respectively. The term ${\tilde{I}}_{\alpha }$ corresponds to the interference modulation pattern, where *α* is the phase delay between the signal and the reference signal. With two settings of *α*, the weight distribution function versus radius *r* (see Figure 19B) of each OAM component can be determined by

(23) $$O_{n}=\frac{1}{4\pi A_{\text{ref}}^{*}}\int_{0}^{2\pi }d\varphi \left({\tilde{I}}_{0}-i{\tilde{I}}_{\pi /2}\right)\exp\left(-in\varphi \right)\text{.}$$

Subsequently, the weight *C*_*n*_ can be calculated by

(24) $$C_{n}=\int_{0}^{\infty }drr{\left|O_{n}\right|}^{2}/\sum_{n}{\int_{0}^{\infty }drr\left|O_{n}\right|}^{2}$$

#### 5.2.3 OAM correlations

**Figure 20:**
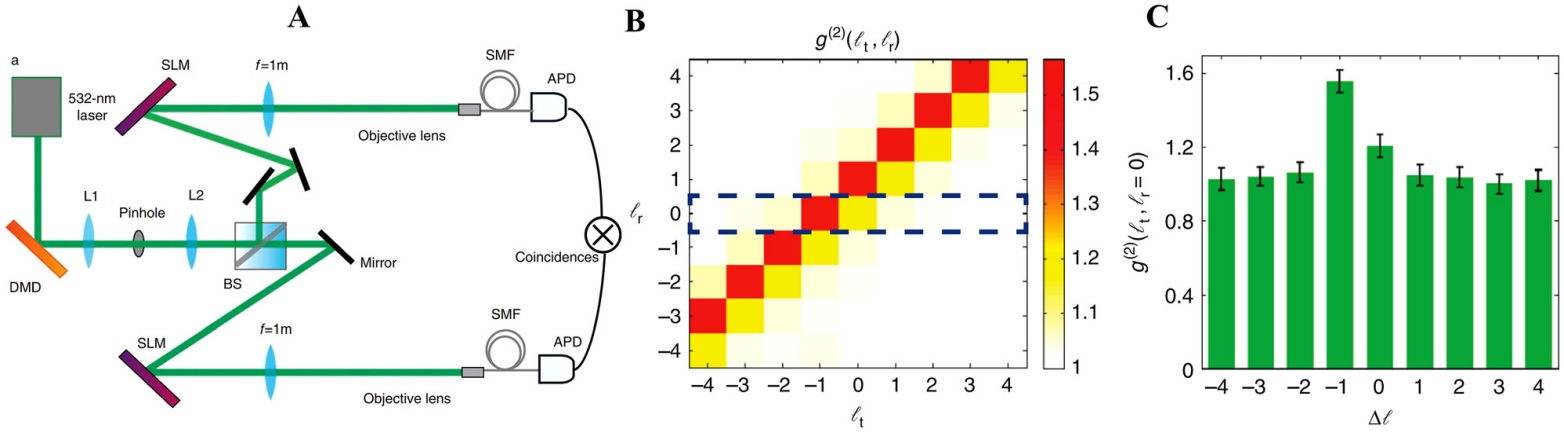
Measurement of the OAM spectrum based on OAM correlations. (A) Experimental setup for digital spiral object identification with random light. (B) Experimental results for the second-order OAM correlation matrix of TC = −2/3. (C) A plot of the row denoted by the dotted box in (B). Reprinted from Ref. [163].

As one of the applications of OAM, object identification can be performed using quantum-correlated OAM states. Similarly, the OAM correlation can also facilitate the calculation of the OAM spectrum of the object, that is, the input fractional vortex beam [163]. In Figure 20A, a pseudothermal light is generated using digital micro-mirror devices (DMD), and such fluctuations of light can result in the formation of intensity correlations in the OAM components. The light source is divided into two arms, one of which is the reference arm, where the SLM is used to load a series of fork grating holograms. The other is a test arm where the SLM is utilized to add another series of fork grating onto the sample to be tested. The normalized second-order OAM correlation function is defined as follows:

(25) $$g^{\left(2\right)}\left(l_{t},l_{r}\right)=\frac{〈I_{l_{t}}I_{l_{r}}〉}{〈I_{l_{t}}〉{〈I_{l_{r}}〉}^{\prime }}\text{,}$$

where 〈I〉 is proportional to the count rate, and $〈I_{l_{t}}I_{l_{r}}〉$ is proportional to the coincidence count rate. *l*_*t*_ represents the test arm, and *l*_*r*_ denotes the reference arm. A two-dimensional matrix with a normalized second-order correlation function is shown in Figure 20B. The 1D plot of the correlation values versus TC of the reference arm facilitates identifying the fractional TC in the test arm, as shown in Figure 20C. This method is based on quantum correlation; thus, it is less sensitive to the coherence properties of the source and robust to turbulence.

Apart from the aforementioned classic methods, there are other methods for measuring the fractional vortex beam. In 2018, Hu et al. proposed a measurement method for integer and fractional vortex beams via the phase-shifting digital holography technique, where the maximal error of the experimental results was less than 4.8% [165]. Moreover, it was verified that the limited integer component can be used to determine the fractional TC [166, 167]. For a perfect fractional vortex beam, the fractional TC can be determined based on the interference of the ±1 order [145] or from the laser speckle [58]. The fractional part of the TC was measured with a high precision (better than 0.01) by illuminating the vortex beam into the edge region of a transparent plate [168]. To summarize, the measurement of fractional vortex beams, particularly the fractional TC, is gradually becoming quantified, and the influence of turbulence and coherence are addressed. In addition to interference and diffraction, some new physical effects have also been explored for the measurement of fractional vortex beams.

## 6 Applications of fractional vortex beam

### 6.1 Optical tweezers

**Figure 21:**
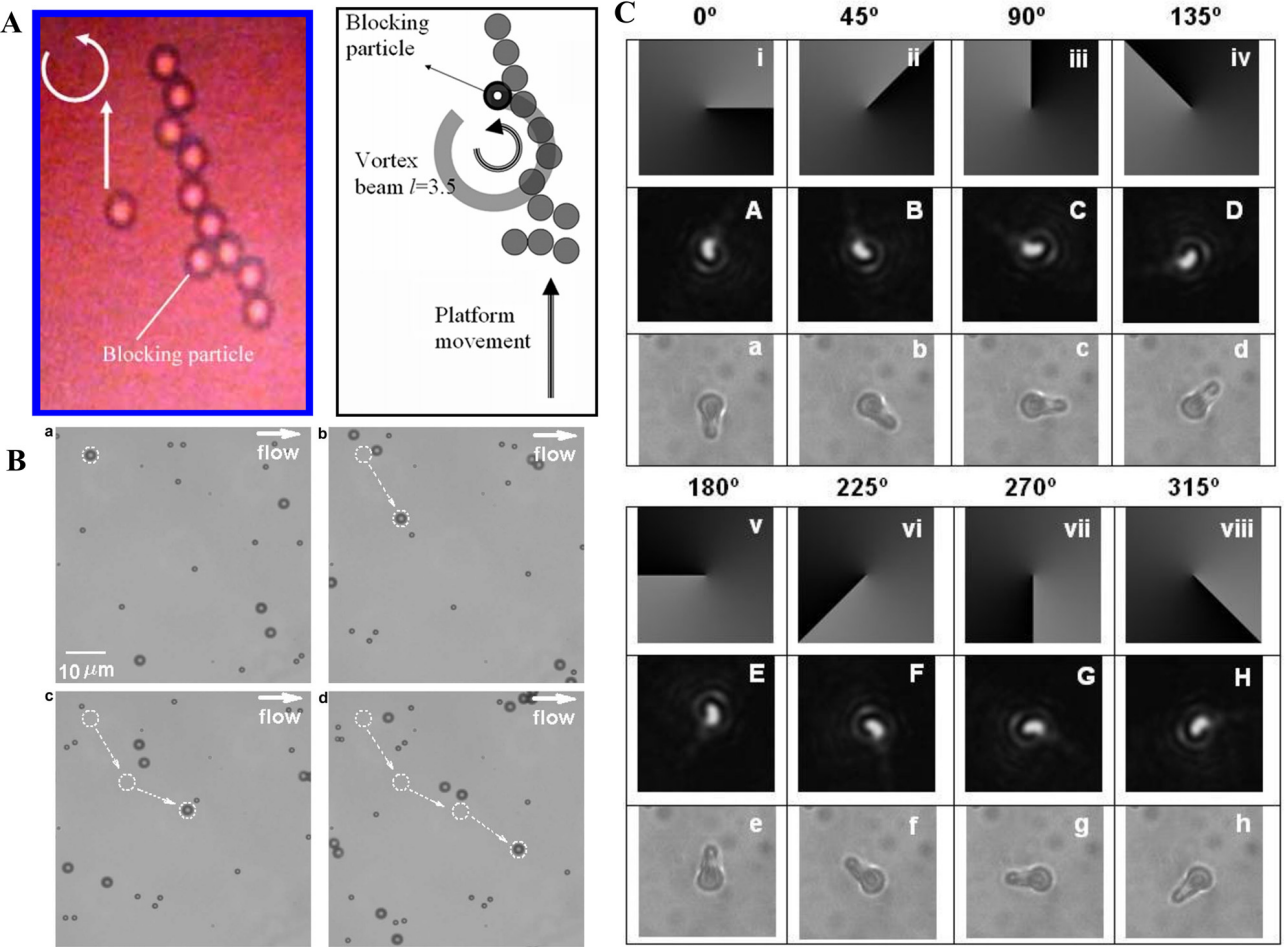
Rotation of trapped red blood cells with fractional vortex beam. (A) Fractional vortex with TC = 3.5 for aligning and transporting particles and the schematic explaining the mechanism. Reprinted from Ref. [63]. (B) Particles trapped by the fractional vortex array, the motion time interval flow speed and laser power are 1 s, 15 μm/s and 2 W, respectively. Reprinted from Ref. [107]. (C) Computer-generated holograms and the trapped red blood cells by the fractional vortex beam with TC = 0.5. Reprinted from Ref. [64].

It is well-known that the OAM carried by a vortex beam can be transferred to microparticles through the interaction between light and matter that results in an angular force; this has attracted extensive attention for application in optical tweezers, particle manipulation, and biology. Compared with the integer vortex beam, the fractional vortex beam has a radial opening (low-intensity gap) in the annular light ring that enables complex manipulation requirements. Similarly, the fractional vortex beam also carries OAM that can be transferred to particles, guiding and transporting them, as shown in Figure 21A [63]. Optical manipulation and sorting by fractional vortex beams have been widely studied. In theory, the energy flow of a vortex beam carrying a pair of fractional vortices was investigated, where the two fractional vortices could be connected by a dark intensity line [169]. To further improve the optical sorting in the experiment, a fractional vortex array that possesses multiple fractional vortices was generated using a phase-only Talbot array illuminator [107]. As demonstrated in Figure 21B, it can be used to improve the properties of optical sorting via the cooperation of two forces (that is, intensity-gradient force and phase-gradient force) and the appropriate direction and flow velocity of the fluids. In addition, the diffraction-free nonparaxial fractional BG vortex was proven to induce spin reversal of an absorptive Rayleigh sphere [170]. In biology, the integer vortex beam can only realize the rotation of cells that limits its application. Favorably, as shown in Figure 21C, the red blood cells trapped by the fractional vortex beam rotate with the intensity gap owing to the OAM transfer. Thereafter, by controlling the rotation angle of the holograms, the angle of the red blood cell can be controlled that may be useful for polarized Raman spectroscopic measurements [64].

### 6.2 Optical communications

**Figure 22:**
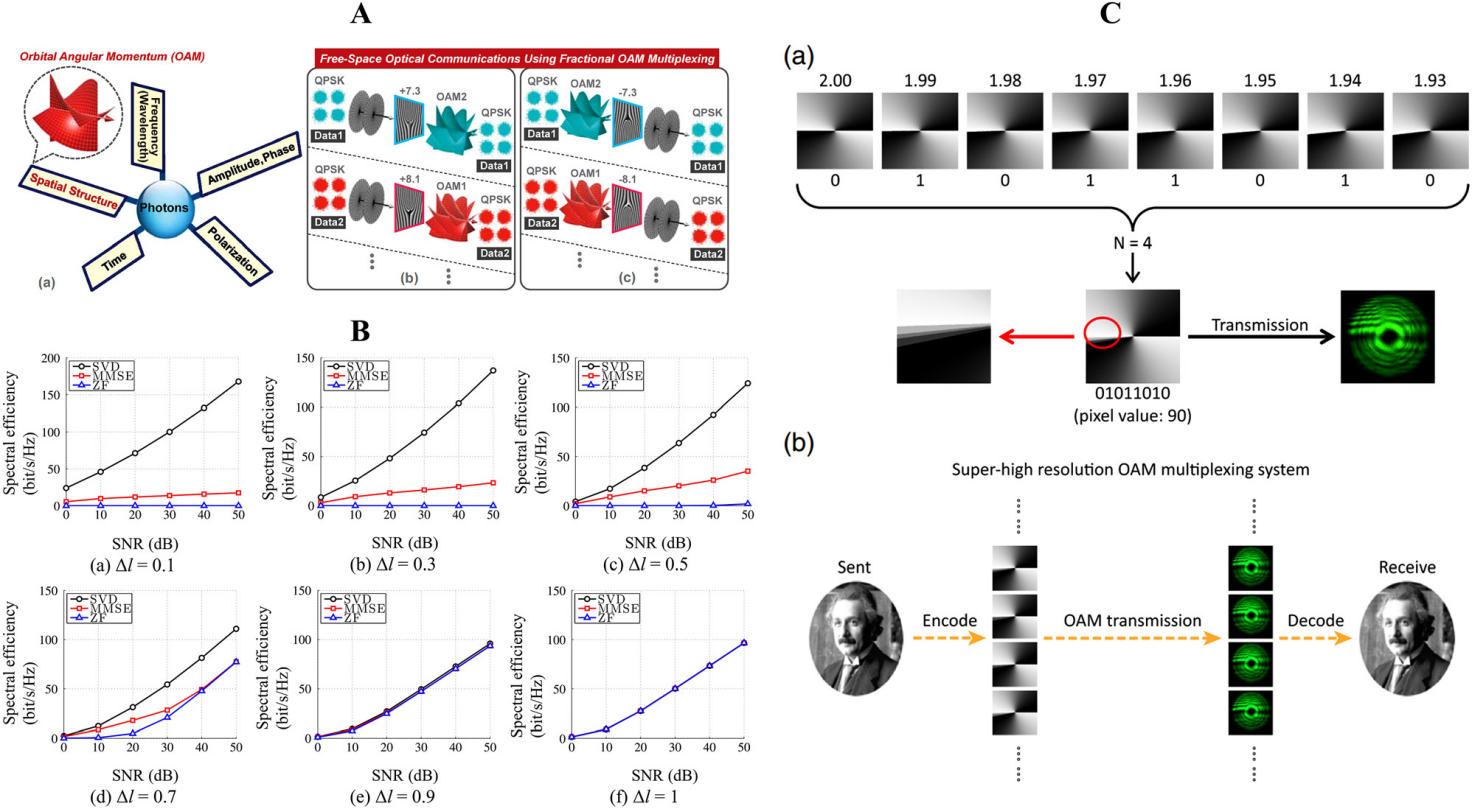
Optical communication based on fractional vortex beam. (A) Basic physical dimensions of freedom of photons and the fractional vortex beam generation and back conversion concept. Reprinted from Ref. [171]. (B) Spectral efficiencies of different detectors and different channel interval. Reprinted from Ref. [172]. (C) OAM superstate multiplexed demonstration encoded by fractional vortex beam and detailed process of transmitting an Einstein portrait. Reprinted from Ref. [158].

In free-space optical communication systems, apart from the conventional physical dimensions (such as amplitude, phase, polarization, time, and frequency), an additional degree of freedom (spatial structure) is explored to increase the communication capacity via space-division multiplexing. The space-division multiplexing-based vortex beam with a spiral phase structure is termed as OAM multiplexing. Hence, multiple OAM states as different carriers can be used for multiplexing and transmitting multiple data streams that provide high degrees of freedom and can be used to realize a significant improvement in communication capacity [65]. The traditional method utilizes OAM beams with integer TC. However, the radius of the vortex beam increases with increasing TC that limits the communication capacity with a limited aperture. Favorably, fractional vortex beams have multiple OAM modes and can be used to address the explosive growth of communication requirements. In 2014, fractional vortex beam communications with atmospheric turbulence were proposed. Assisted by multiple-input multiple-output equalization for effectively mitigating turbulence-induced crosstalk, it realized a robust fractional vortex communication, where the TC was half-integer [66]. The OAM channel interval of the aforementioned OAM-based optical communications was one. Subsequently, the OAM channel interval was reduced to less than one; this is also known as fractional OAM multiplexing [171]. In this work, free-space optical communications based on the fractional vortex beam with the smallest OAM channel interval of 0.6 was successfully realized, as shown in Figure 22A. However, owing to the significant mode crosstalk, the performance degraded correspondingly with a decreased channel interval. To further increase the capacity, an arbitrary-order OAM multiplexing system with a channel interval of less than 0.5 was proposed, in which the spectral efficiency was increased with a decrease in the channel interval of the adjacent OAM modes, as shown in Figure 22B [172].

However, the interval between adjacent OAM modes cannot be extremely small because of the limit of the system resolution. Hence, precisely recognizing the OAM modes in the receiving end plays a crucial role in communication capacity expansion. Recently, based on the method of deep learning, the minimum interval recognized between adjacent OAM modes was decreased to 0.01 to realize a high-resolution identification of fractional TCs and unlimitedly expand the communication capacity in theory [158]. As depicted in Figure 22C, an 8-bit code composed of eight different OAM modes with a TC from 1.93 to 2.00 only occupies a small region in the intensity of the superstate that indicates that a large capacity can continuously be used for encoding. An Einstein portrait was used to verify the performance of the system. It has potential applications for OAM multiplexing with the smallest mode interval of 0.01 and <0.02% bit error rate. Subsequently, Zhu et al. proposed the ultra-dense perfect OAM holography with a TC resolution of 0.01, where the OAM modes are multi-dimensional in radial and angular [67]. Furthermore, the optical communication properties of the fractional vortex beam in an underwater system and LED optical system were investigated [173, 174]. In addition, OAM modes also can be used in fiber optical communication system, and the methods such as multiple-input-multiple-out equalization, adaptive optics and novel fibers are proposed to supporting the integer OAM modes [175, 176]. For fractional OAM modes, Alexeyev et al. theoretically demonstrate that in circular arrays of anisotropic fibers at certain distribution of anisotropy directors robust transmission of fractional vortex with half-integer TCs is possible [177]. These studies verified that the nonorthogonal signal is also one of the candidate methods that could address the rapidly increasing demand for communication capacity.

### 6.3 Optical imaging

**Figure 23:**
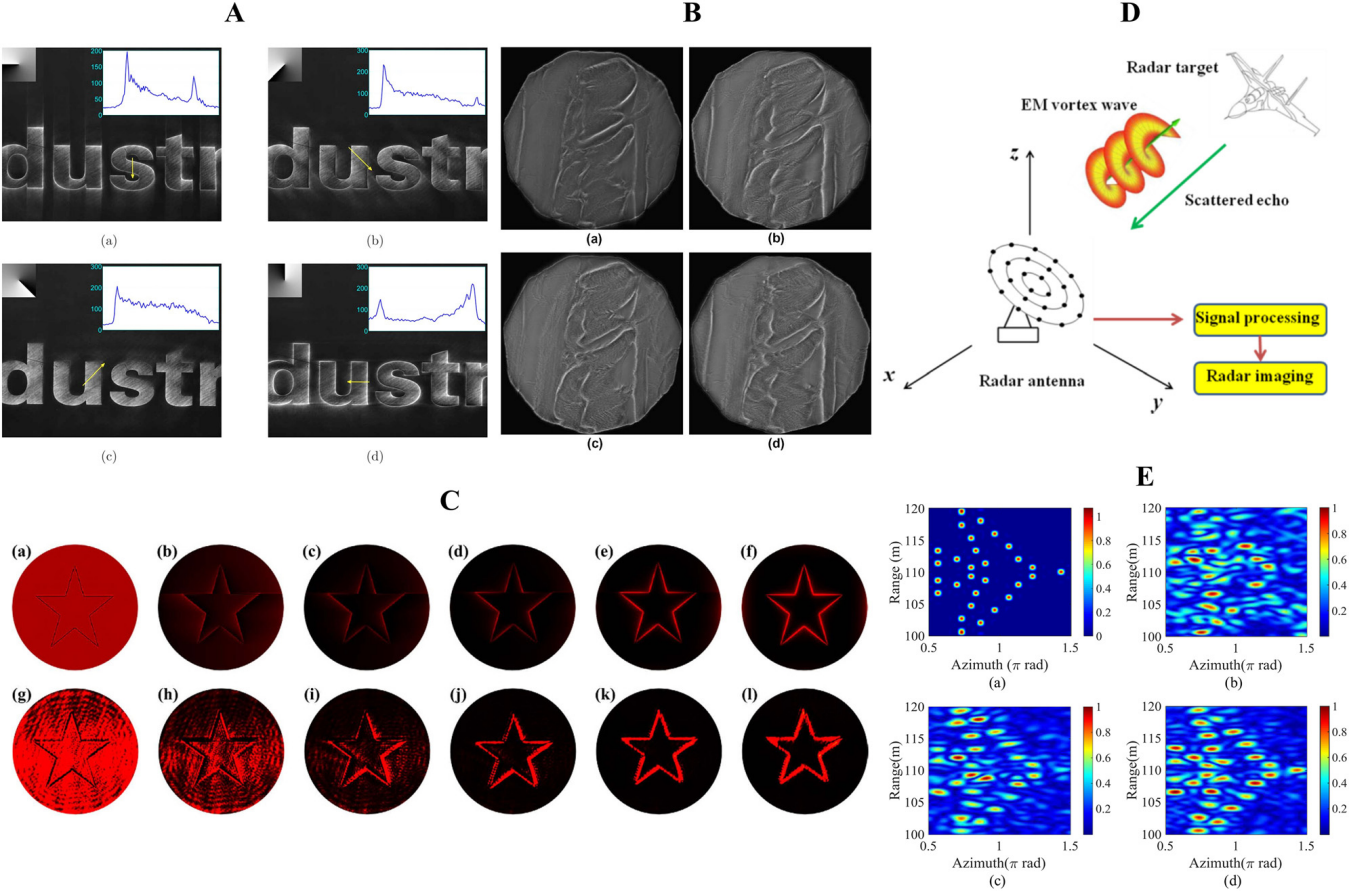
Applications of the fractional vortex beam in optical imaging field. (A) Experimental results of the edge enhancement of a transparent object “dustr” based on a fractional SPP. Reprinted from Ref. [70]. (B) Edge enhancement of microtome taste buds from a rabbit. Reprinted from Ref. [185]. (C) Simulation (a–f) and experimental (g–l) results of an edge enhancement version of a five-pointed star pattern where the values of TC are (a, g): 0, (b, h): 0.2, (c, i): 0.4, (d, j): 0.6, (e, k): 0.8, and (f, l): 1, respectively. Reprinted from Ref. [186]. (D) Schematic of the electromagnetic vortex imaging based on radar imaging system. Reprinted from Ref. [191]. (E) Result images obtained with different OAM sampling intervals and signal-to-noise ratio of 5 dB, (a) Ground truth. (b) Δ*l* = 1. (c) Δ*l* = 0.5. (d) Δ*l* = 0.2. Here, Δ*l* means the interval between two OAM modes. Reprinted from Ref. [72].

In the field of optical imaging, the vortex beam has been used in spiral phase contrast imaging technology that plays an important role in image processing [178] and edge enhancement of optical microscopy [179]. In the field of spiral phase contrast imaging, the integer SPP has a radial symmetric phase structure and results in an orientation-independent edge-enhancement of the input image that implies that all ranges of images are edge-enhanced [180], [181], [182]. The progress of the spiral phase filtering can be regarded as a convolution of the input image and the Fourier transform of the SPP. In contrast to spiral phase filtering with integer TC, fractional spiral phase filtering can realize edge enhancement based on controllable degree and orientation of enhancement along the perpendicular orientation of the edge discontinuity line in the SPP [61, 71, 183], [184], [185], [186], [187]. As illustrated in Figure 23A, the orientation values of the phase discontinuity are π, 5π/4, 7π/4, and π/2, and the values of TC are 0.8, 0.6, 0.4, and 0.9, respectively [70]. The curves show the edge enhancement of the image along the yellow line that indicates that a high-contrast edge enhancement will be obtained with TC > 0.5, and low edge enhancement with TC < 0.5, owing to the absence of a vortex. Subsequently, edge enhancement based on fractional SPP was produced via optical microscopy [185]. The specimen was the taste buds of a rabbit, and the degree of edge enhancement increased with increasing TC, as demonstrated in Figure 23B. However, the object was complex and edge enhancement could not be observed clearly. Further, as depicted in Figure 23C, researchers use an SLM to generate a fractional SPP and another SLM to display a simple phase object with a five-pointed star that exhibits an evident and controllable edge enhancement of the image [186].

In addition, electromagnetic waves with OAM have potential applications in 2D radar staring imaging owing to the relationship between the azimuthal angle and the OAM mode [188], [189], [190]. The multiple-in-multiple-out system possesses a higher azimuthal resolution than the multiple-in-single-out system that is related to the range of the OAM modes. However, the scattering points could not be correctly separated when the azimuthal angle difference was larger than 180° that caused an aliasing problem. To address this problem, researchers utilized an equivalent fractional OAM mode (the OAM mode refers to two adjacent integers for receiving and transmitting ends) to realize high resolution. A schematic of the electromagnetic vortex wave-based radar imaging system is shown in Figure 23D [191]. Subsequently, the simulation results based on the Monte Carlo method verified that the fractional vortex imaging method exhibited better imaging performance in a low signal-to-noise ratio environment. As shown in Figure 23E(b–d), the signal-to-noise ratio is −5 dB, and the intervals between the two OAM modes are 1, 0.5, and 0.2, respectively. Figure 23E(a) is a target object, and it can be observed that a better image enhancement ability of the aircraft can be achieved with a smaller difference between the values of the two OAM modes at the receiving and transmitting ends [72]. Hence, the fractional vortex beam can realize an anisotropy edge enhancement imaging by breaking down the symmetry of the filtering process, and higher resolution imaging owing to the smaller OAM interval.

### 6.4 Quantum entanglement

**Figure 24:**
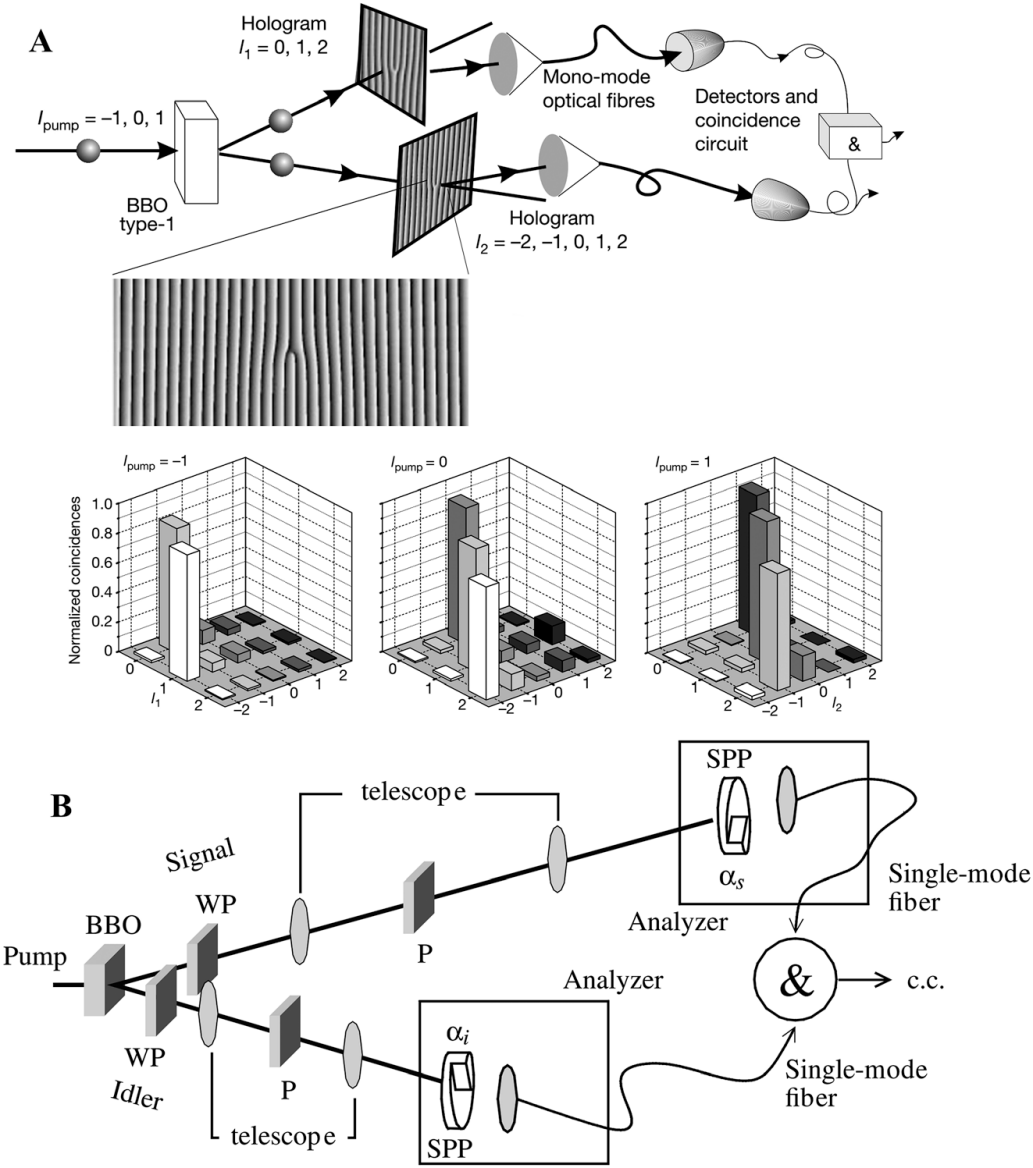
Applications of the fractional vortex beam in high-dimensional quantum entanglement. (A) Integer SPP analyzers. Reprinted from Ref. [192]. (B) Fractional SPP analyzers. Reprinted from Ref. [73].

The twin photons generated with an ideal spontaneous parametric down-conversion process are entangled in the full-and infinite-dimensional Hilbert space. In 2001, Mair et al. [192] experimentally demonstrated OAM entanglement using integer-OAM analyzers (see Figure 24A). A few years later, Oemrawsingh et al. realized ultra-high-dimensional entanglement using fractional SPP analyzers [73]. As shown in Figure 24B, a fractional-OAM analyzer with TC = 3.48 is set in the signal path and an analyzer with SPP index TC = −3.48 is placed in the idler path. The parabolic photon coincidence fringes are expected to be a function of only the relative orientation of the two SPPs that reveals the entanglement of the twin photons. They realized a *D* > 3700 per photon entanglement that limited the entangled modes emitted by the nonlinear crystal.

## 7 Conclusions and perspectives

Light beams carrying fractional TC have significantly improved the control of the degree of freedom of vortex beams, leading to new perspectives in optics. In this review, we briefly review the recent research process in the field of fractional vortex beam theory, propagation, generation, measurement, and applications. Six models of fractional vortex beams were proposed based on the modification of their amplitude and phase distribution. The propagation stability of fractional vortex beams can be significantly improved by combining nondiffraction characteristics of BG beams or by controlling the number of integer-order vortex modes for mode superposition. Furthermore, the fractional TC, as an important parameter, assumes the nearest integer value and changes as the transmission distance varies. The fractional vortex beam can be generated by conventional diffractive elements for modifying their propagation phase or exploiting a metasurface to combine the control of the geometric phase and retardation phase with the trend of compactness and high integration. Methods based on diffraction, interference, and machine learning can achieve high-precision measurement of the TC and OAM spectrum of fractional vortex beams. Owing to their extraordinary properties, fractional vortex beams are useful in many optical applications, such as realizing cell sorting in optical tweezers, improving the communication capacity in optical communication systems, realizing image edge enhancement and high resolution in optical imaging fields, and high-dimensional quantum entanglement.

However, the existing research on fractional vortex beams is not sufficient. The fractional phase step implies that a more specific fractional vortex can be designed by modulating the spiral phase based on various distributions. Furthermore, high-order fractional vortices with high mode purity are difficult to generate owing to the diffraction effect that is a significant challenge for the future. To further facilitate the applications of fractional vortex beams, more metasurfaces with specific structures should be designed to facilitate applications at the nanoscale as well. In the future, more flexible and efficient metasurfaces will be developed to generate fractional vortex beam with various amplitude and phase distributions, which will help to realize more sophisticated optical manipulation. Over the past few decades, most of studies on fractional vortex beams have been focused on the fully coherent light field, but the new physical effects induced by the modulation of coherence on the light field are non-negligible. The various types of fractional vortex beams with controllable spatial coherence characteristics improve the modulation degree of freedom and may have a meaningful effect in practical applications. The resolution capability of fractional OAM modes plays a crucial role in optical communication. A carefully developed metasurface at the nanoscale has a higher resolution that could be a candidate for precisely measuring the fractional TC. In addition, the specific transmission distance and information capacity of the fractional vortex beam communication and the possibility of application in fiber optical communication should be studied in the future. Furthermore, vortices with fractional TC can also be extended to acoustics and electron beams, which may be useful for physics, biosciences and engineering as well.
